# GPIHBP1, lipoprotein lipase, and triglyceride-rich lipoproteins in capillaries of the choroid plexus and circumventricular organs

**DOI:** 10.1172/JCI191867

**Published:** 2025-10-01

**Authors:** Wenxin Song, Madison Hung, Ellen Kozlov, Megan Hung, Anh P. Tran, James Carroll, Le Phoung Nguyen, Troy L. Lowe, Paul Kim, Hyesoo Jung, Yiping Tu, Joonyoung Kim, Ashley M. Presnell, Julia Scheithauer, Jenna P. Koerner, Ye Yang, Shino D. Magaki, Christopher K. Williams, Michael Ploug, Haibo Jiang, Christer Betsholtz, Maarja Andaloussi Mäe, Liqun He, Anne P. Beigneux, Loren G. Fong, Stephen G. Young

**Affiliations:** 1 Department of Medicine,; 2 Department of Human Genetics, and; 3 Department of Pathology and Laboratory Medicine, David Geffen School of Medicine, UCLA, Los Angeles, California, USA.; 4 Finsen Laboratory, Copenhagen University Hospital - Rigshospitalet, Copenhagen N, Denmark.; 5 Biotechnology Research and Innovation Centre (BRIC), University of Copenhagen, Copenhagen N, Denmark.; 6 Department of Chemistry, The University of Hong Kong, Hong Kong, China.; 7 Department of Immunology, Genetics, and Pathology, Rudbeck Laboratory, Uppsala University, Uppsala, Sweden.; 8 Department of Medicine-Huddinge, Karolinska Institute Campus Flemingsberg, Huddinge, Sweden.

**Keywords:** Metabolism, Vascular biology, Endothelial cells, Lipoproteins

## Abstract

In peripheral tissues, an endothelial cell (EC) protein, GPIHBP1, captures lipoprotein lipase (LPL) from the interstitial spaces and transports it to the capillary lumen. LPL mediates the margination of triglyceride-rich (TG-rich) lipoproteins (TRLs) along capillaries, allowing the lipolytic processing of TRLs to proceed. TRL-derived fatty acids are used for fuel in oxidative tissues or stored in adipose tissue. In mice, GPIHBP1 is absent from capillary ECs of the brain (which uses glucose for fuel); consequently, LPL and TRL margination are absent in mouse brain capillaries. However, because fatty acids were reported to play signaling roles in the brain, we hypothesized that LPL-mediated TRL processing might occur within specialized vascular beds within the central nervous system. Here, we show that GPIHBP1 is expressed in capillary ECs of human and mouse choroid plexus (ChP) and that GPIHBP1 transports LPL (produced by adjacent ChP cells) to the capillary lumen. The LPL in ChP capillaries mediates both TRL margination and processing. Intracapillary LPL and TRL margination are absent in the ChP of *Gpihbp1*^–/–^ mice. GPIHBP1 expression, intracapillary LPL, and TRL margination were also observed in the median eminence and subfornical organ, circumventricular organs implicated in the regulation of food intake.

## Abstract 2

The brain uses glucose for fuel, but we discovered that triglyceride-rich lipoproteins undergo lipolytic processing in specialized vascular beds within the central nervous system.

## Graphical abstract

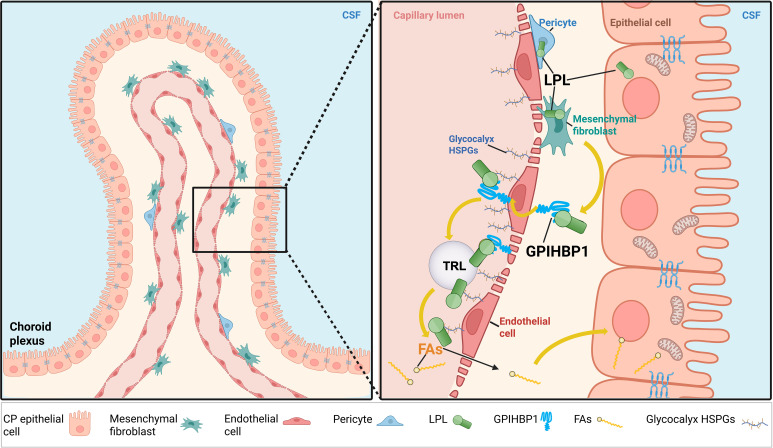

## Introduction

The intravascular lipolytic processing of triglyceride–rich (TG-rich) lipoproteins (TRLs) by lipoprotein lipase (LPL) is crucial for delivering dietary lipids to oxidative tissues (e.g., the heart) for fuel and to white adipose tissue for storage (1). In mammals, LPL is synthesized mainly by myocytes and adipocytes (2) and then secreted into the surrounding interstitial spaces (3, 4). The interstitial LPL is then captured by GPIHBP1, an endothelial cell (EC) protein, and transported to the luminal surface of capillaries (3). LPL mediates both the margination of TRLs along capillaries and the hydrolysis of TGs in TRL particles (5). A tryptophan-rich loop in LPL’s carboxyl-terminal domain (6) is required for both TRL margination and TG hydrolysis (5, 7, 8). In *Gpihbp1*^–/–^ mice, the LPL produced by myocytes and adipocytes is stranded within the interstitial spaces and never reaches the capillary lumen, resulting in impaired TRL processing and severe hypertriglyceridemia (3).

GPIHBP1 is solely responsible for transporting LPL to the luminal surface of capillary ECs (3), but a substantial amount of the LPL (~ 25%) that is moved into capillaries detaches from GPIHBP1 and is captured by heparan sulfate proteoglycans (HSPGs) within the glycocalyx of capillary ECs (7). Confocal micrographs, immunogold electron micrographs, and NanoSIMS imaging have documented LPL in the glycocalyx of heart capillary ECs (distant from the GPIHBP1 on the luminal plasma membrane of ECs) (7).

NanoSIMS analyses of heart and brown adipose tissue have made it possible to visualize the margination of stable isotope–enriched TRLs within capillaries, the lipolytic processing of those TRLs, and the trafficking of TRL-derived fatty acids into adjacent parenchymal cells (9–12). In one such study, WT mice were given an intravenous (IV) injection of [^2^H]TRLs (11). After either 30 seconds or 2 minutes, the mice were euthanized; the vasculature was perfused extensively; and sections of the heart were prepared for NanoSIMS analyses of elemental and isotopic composition. ^2^H/^1^H NanoSIMS images of heart sections documented margination of [^2^H]TRLs along heart capillaries and abundant [^2^H]TRL-derived lipids in the mitochondria and cytosolic lipid droplets of cardiomyocytes (11). Thus, the lipid products of intracapillary TRL processing move into parenchymal cells extremely rapidly. In *Gpihbp1*^–/–^ mice (where intracapillary LPL is absent), the margination of [^2^H]TRLs along heart capillaries, and the entry of [^2^H]TRL-derived lipids into cardiomyocytes were markedly reduced (11).

The brain uses mainly glucose (rather than TRL-derived fatty acids) for fuel. Also, the microvasculature of the brain is characterized by a blood-brain barrier (BBB) (formed by capillary ECs with tight junctions, pericytes, and astrocyte endfeet) that regulates nutrient exchange between the systemic circulation and the brain parenchyma. During development, a lysophosphatidylcholine transporter, MFSD2A, is crucial for transporting essential fatty acids across BBB capillaries and into the brain parenchyma (13–15). *Gpihbp1* transcripts are absent in ECs of the mouse brain parenchyma (16); consequently, LPL is absent from the luminal surface of capillaries (5, 7). Consistent with those observations, we did not observe TRL margination along capillaries in the mouse cerebellum (5). Also, we did not observe [^2^H]TRL margination or processing in the mouse cerebral cortex, whereas the uptake of [^13^C]glucose by the mouse cortex was extremely robust (17). Interestingly, the properties of the glycocalyx in heart capillaries and BBB capillaries appear to be distinct (7). When recombinant human LPL was injected IV into *Gpihbp1*^–/–^ mice, the LPL bound avidly to the glycocalyx of heart capillary ECs, but there was little or no binding to the glycocalyx of BBB capillaries (7).

Even though LPL-mediated TRL processing is virtually absent in BBB capillaries of the mouse (5, 7, 16, 17), several rodent studies have raised the possibility that free fatty acids regulate hypothalamic orexigenic hormone expression and feeding behavior (18, 19). An infusion of oleic acid into the third ventricle of rats resulted in reduced food intake and reduced expression of neuropeptide Y in the hypothalamus (19). These observations were intriguing, but the origin of the fatty acids that would participate in the physiologic regulation of food intake is not clear. Neurons in the brain produce LPL (20–22), and inactivating neuronal *Lpl* expression in mice has been reported to increase expression of orexigenic hormones (agouti-related protein, neuropeptide Y) and trigger obesity (by influencing food intake and activity) (23). Those observations suggested that LPL produced by neurons could be relevant to energy metabolism, but the mechanism is not clear. Neither the location of the LPL secreted by neurons (whether that LPL is intravascular or extravascular) nor the glycerolipid substrates for neuronal LPL has been defined.

While GPIHBP1, intracapillary LPL, and TRL margination are absent in mouse BBB capillaries (5, 7, 17), we have been reluctant to dismiss the possibility of LPL-mediated TRL processing in the CNS. We hypothesized that TRL processing could occur in specialized vascular beds within the CNS. The capillaries of the choroid plexus (ChP) and circumventricular organs (e.g., median eminence, subfornical organ) are fenestrated and lack a BBB (24–26), but, to the best of our knowledge, no one had ever considered the possibility of LPL-mediated intravascular lipolysis in those locations. We were positioned to address this possibility because we have developed methods to assess GPIHBP1 expression in capillaries, amounts of LPL in the capillary lumen, and TRL margination along capillaries (4, 7, 27, 28). In the current studies, we sought to determine whether GPIHBP1 is present in the human and mouse ChP, and if so, whether GPIHBP1 expression is accompanied by intracapillary LPL and TRL margination. In addition to investigating the ChP, we investigated GPIHBP1 expression, intracapillary LPL, and TRL margination in capillaries of the median eminence and subfornical organ, circumventricular organs that have been implicated in the hypothalamic regulation of feeding behavior (29–33).

## Results

### GPIHBP1 and LPL expression in the ChP.

**Figure 1:**
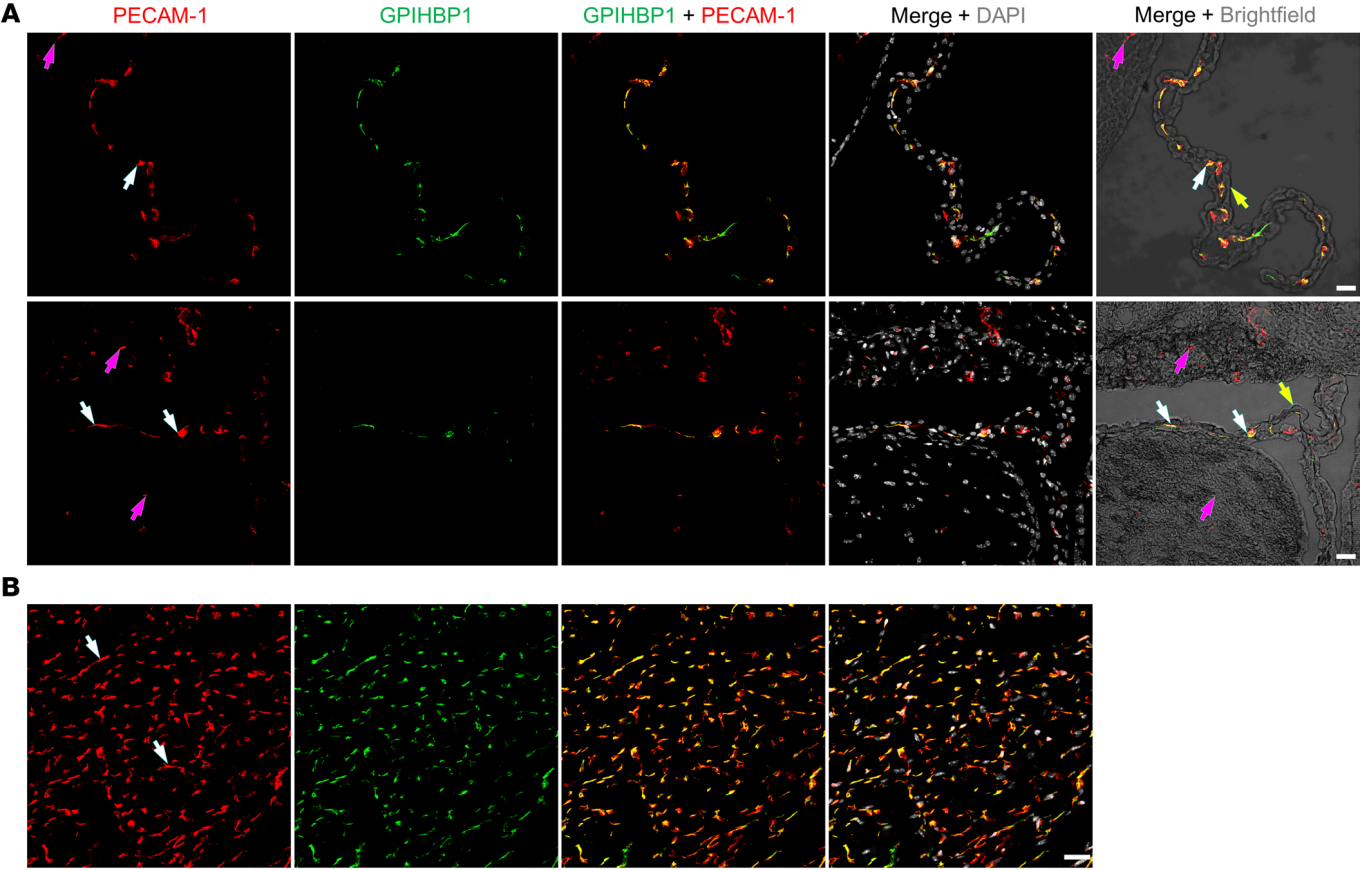
Confocal micrographs revealing GPIHBP1 in PECAM-1–positive capillary ECs of a WT mouse. Sections of choroid plexus (ChP) (**A**) and heart (**B**) were incubated with mAb 11A12 (against GPIHBP1) and a rabbit polyclonal antibody against PECAM-1. The binding of primary antibodies was detected with Alexa Fluor–labeled secondary antibodies. In ChP and heart, mAb 11A12 bound to the PECAM-1–positive capillaries (white arrows) but did not bind to PECAM-1–positive capillaries in the brain parenchyma (purple arrows). Brightfield images made it possible to visualize ChP epithelial cells (yellow arrows). Images were recorded with a 20 × objective. Scale bar: 20 μm.

We used a rat monoclonal antibody (mAb) against mouse GPIHBP1 (11A12) and standard IHC methods to assess GPIHBP1 expression in ChP capillaries of WT mice. Our studies revealed that GPIHBP1 is present in ChP capillary ECs, colocalizing with PECAM-1 (an EC marker protein) (Figure 1 and Supplemental Figure 1; supplemental material available online with this article; https://doi.org/10.1172/JCI191867DS1). PECAM-1 was expressed in capillaries of the brain parenchyma, but GPIHBP1 was absent. As expected, GPIHBP1 and PECAM-1 were present in capillary ECs of the heart (Figure 1 and Supplemental Figure 1). The extent of GPIHBP1 and PECAM-1 colocalization in ChP and heart capillary segments was very strong, as judged by Pearson’s correlation coefficients (Supplemental Figure 2). Consistent with the IHC findings, *Gpihbp1* transcripts were detected in microdissected ChP but were absent in the cerebral cortex (*n* = 6–7 WT mice) (Supplemental Figure 3A). The level of *Gpihbp1* expression in the mouse ChP, relative to *Pecam1* expression, was less than 10% of the level in the heart (Supplemental Figure 3B). As expected, transcripts for transthyretin (*Ttr*) and folate receptor 1 (*Folr1*), which are markers of ChP epithelial cells, were detected in the ChP but not in the cerebral cortex or heart (Supplemental Figure 3C).

**Figure 2:**
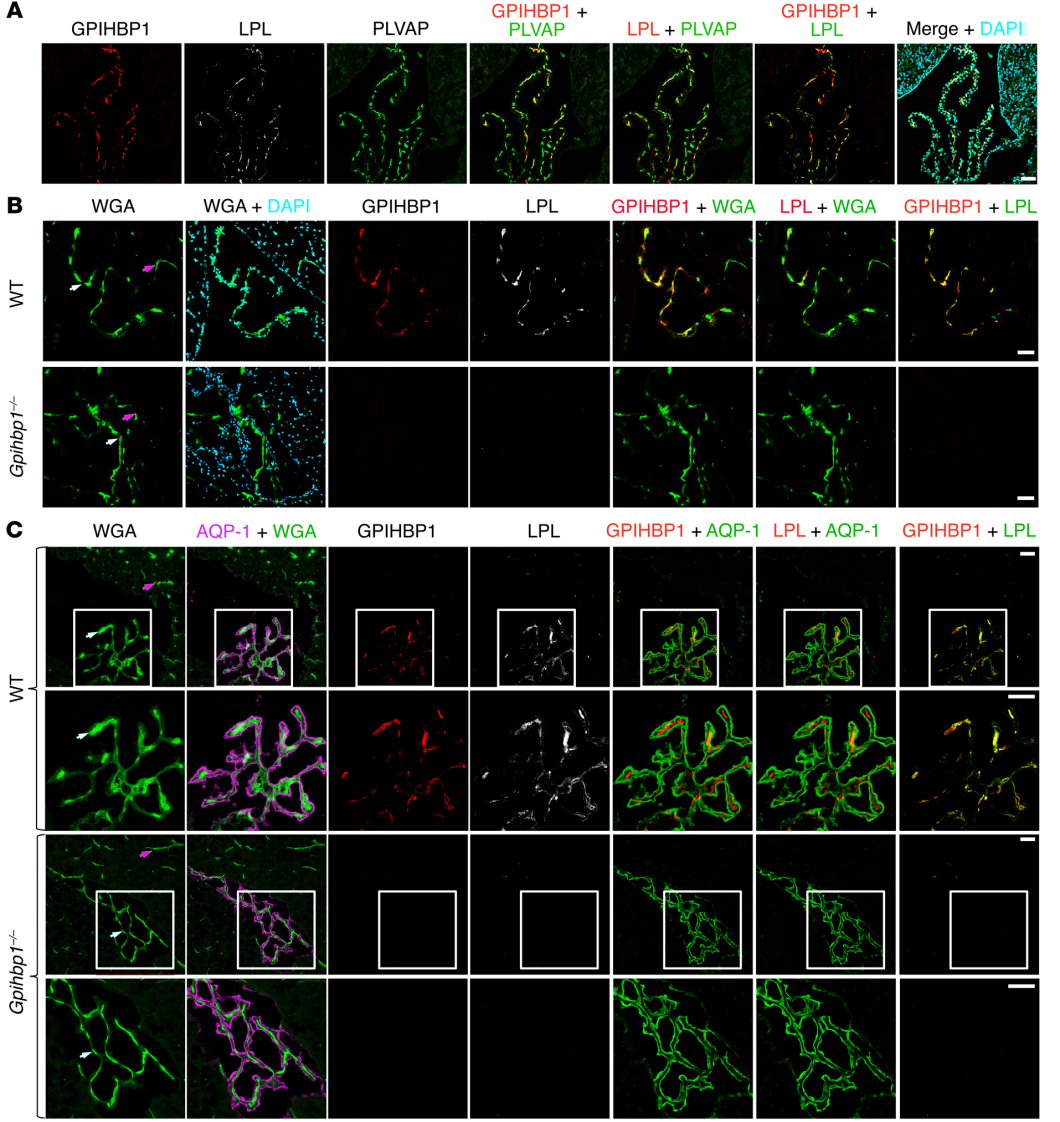
GPIHBP1 and LPL are on the luminal surface of ChP capillaries in WT mice but not *Gpihbp1*^–/–^ mice. (**A**) Confocal micrographs of the ChP. Alexa Fluor 488–MECA-32 (against plasmalemma vesicle-associated protein [PLVAP]; green), Alexa Fluor 555–11A12 (against GPIHBP1; red), and Alexa Fluor 647–27A7 (against LPL; white) were injected IV into WT mice. After 2 min, the vasculature was perfused extensively with PBS and PFA, and tissue sections were prepared for microscopy. DNA was stained with DAPI. mAbs 11A12 and 27A7 bound to the luminal surface of PLVAP-positive ChP capillaries. Scale bar: 50 μm. (**B** and **C**) Confocal micrographs to assess binding of 11A12 and 27A7 to the luminal surface of ChP capillaries in WT and *Gpihbp1^–/–^* mice. Mice were given an IV injection of Alexa Fluor 647–27A7 (white), Alexa Fluor 555–11A12 (red), and Alexa Fluor 488–wheat germ agglutinin (WGA, green). After 2 min, the vasculature was perfused, and tissue sections were prepared for microscopy. In **C**, ChP epithelial cells were identified with an AQP-1–specific mAb followed by Alexa Fluor Plus 405–anti-rabbit IgG (H+L) (purple). GPIHBP1 and LPL were detected along the luminal surface of ChP capillaries in WT mice (white arrows) but not in *Gpihbp1^–/–^* mice. WGA bound to capillaries in the ChP and the brain parenchyma (purple arrows). Boxed images of the ChP in **C** are shown below at higher magnification. Images were recorded with a 20 objective. Scale bar, 50 μm.

To determine if GPIHBP1 and LPL were located on the luminal surface of ChP capillaries, we gave WT mice an IV injection of mAb MECA-32 (against PVLAP, a marker of fenestrated ECs), mAb 11A12 (against GPIHBP1), and mAb 27A7 (against LPL), each conjugated to an Alexa Fluor dye. After 2 minutes, the mice were euthanized; the vasculature was perfused extensively with PBS; and tissue sections were prepared for microscopy. All 3 mAbs bound to the luminal surface of ChP capillaries (Figure 2A). We also gave WT and *Gpihbp1*^–/–^ mice an IV injection of mAbs 11A12, 27A7, and wheat germ agglutinin (WGA), each labeled with an Alexa Fluor dye. WGA binds to HSPGs on the luminal surface of ECs. After 2 minutes, the vasculature was perfused, and tissue sections were prepared for microscopy. 11A12 and 27A7 bound to the luminal surface of ChP capillaries in WT but not *Gpihbp1*^–/–^ mice (Figure 2B), demonstrating that the presence of LPL inside capillaries depends on GPIHBP1. In other experiments, WT and *Gpihbp1*^–/–^ mice were given an IV injection of mAbs 11A12 and 27A7. We then perfused the vasculature with PBS, prepared tissue sections, and stained sections with a mAb against AQP-1 (a marker of ChP epithelial cells) (Figure 2C). In WT mice, the GPIHBP1 and LPL mAbs bound to WGA-positive capillaries (which were surrounded by AQP-1–positive ChP epithelial cells). In *Gpihbp1*^–/–^ mice, WGA-positive capillaries were surrounded by ChP epithelial cells, but there was no binding of the GPIHBP1 or LPL mAbs to the luminal surface of capillaries (Figure 2C).

In a control experiment, we gave WT mice an IV injection of mAb 2H8 (against PECAM-1) and 1 mouse LPL-specific antibodies (mAb 27A7 and the rabbit polyclonal antibody Ab3174), each labeled with an Alexa Fluor dye. The vasculature was perfused extensively with PBS, and tissue sections were prepared for microscopy. Both LPL-specific antibodies bound to the luminal surface of ChP capillaries; mAb 2H8 (against PECAM-1) bound to both ChP capillaries and capillaries in the surrounding brain parenchyma (Supplemental Figure 4). In another control experiment, we assessed binding of mAb 27A7 (against mouse LPL) and mAb 5D2 (against human LPL) to the luminal surface of ChP capillaries in WT mice and *Lpl*^–/–^MCK-hLPL mice (*Lpl* knockout mice that express human LPL from a muscle-specific promoter) (34). mAb 27A7, but not 5D2, bound to the luminal surface of ChP capillaries of WT mice; neither mAb bound to ChP capillaries in *Lpl*^–/–^MCK-hLPL mice (Supplemental Figure 5A). mAb 27A7, but not 5D2, bound to heart capillaries of WT mice; mAb 5D2, but not 27A7, bound to heart capillaries of *Lpl*^–/–^MCK-hLPL mice (Supplemental Figure 5B). These studies confirmed the species specificity of the LPL mAbs and demonstrated that the perfusion of the vasculature with PBS was effective in removing unbound mAbs.

### Lpl and Gpihbp1 expression in the ChP.

**Figure 3:**
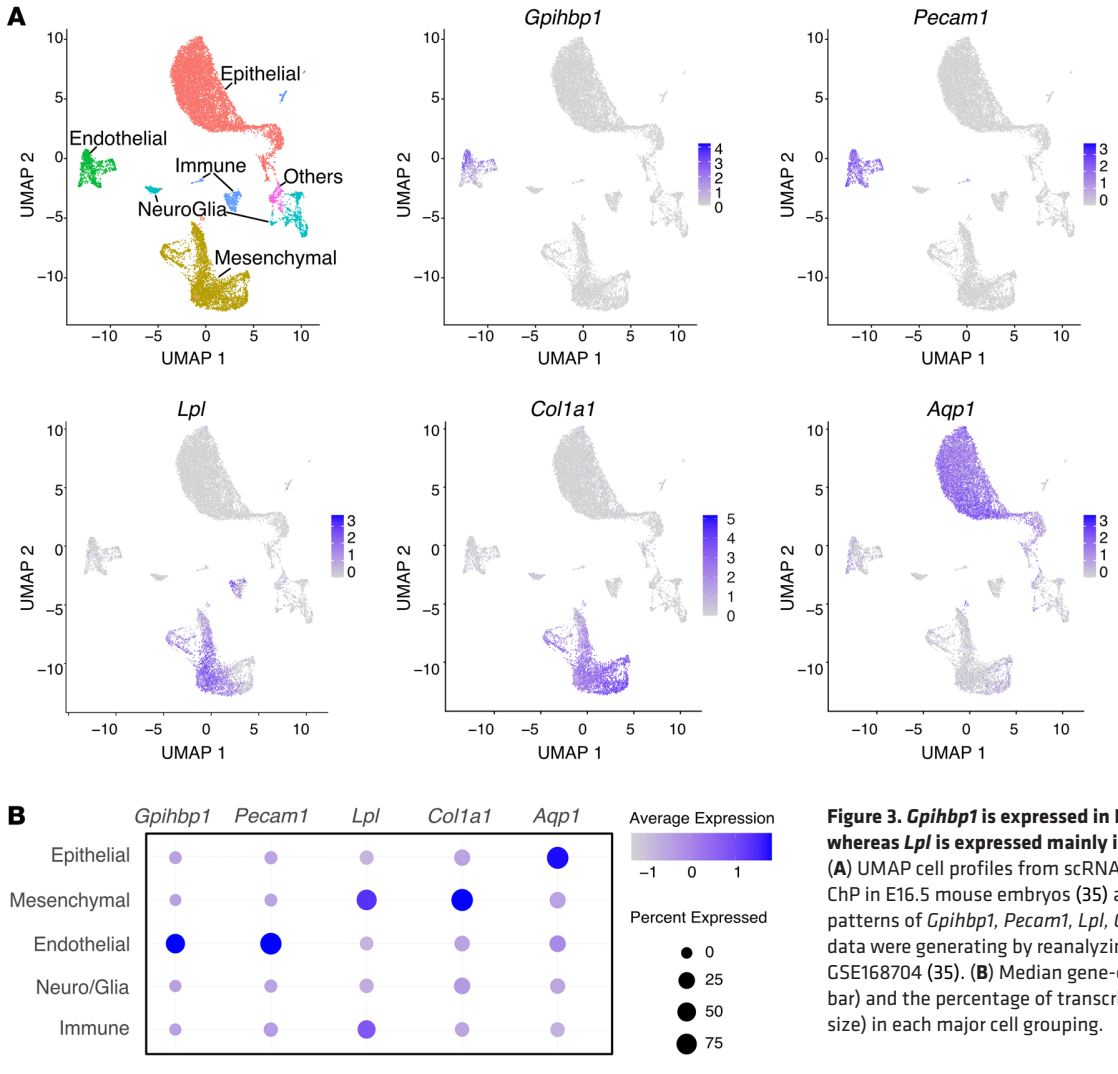
*Gpihbp1* is expressed in ECs of the ChP, whereas *Lpl* is expressed mainly in mesenchymal cells. (**A**) UMAP cell profiles from scRNA-seq studies of the ChP in E16.5 mouse embryos (35) along with expression patterns of *Gpihbp1, Pecam1, Lpl, Col1a1,* and *Aqp1*. These data were generating by reanalyzing the dataset in GEO: GSE168704 (35). (**B**) Median gene-expression level (color bar) and the percentage of transcript-positive cells (circle size) in each major cell grouping.

We used single-cell RNA-seq (scRNA-seq) data (35) from the mouse ChP to define the location of *Gpihbp1* and *Lpl* expression. *Gpihbp1* transcripts were in *Pecam1*-positive ECs; *Lpl* transcripts were located mainly in *Col1a1-*positive mesenchymal fibroblasts; *Aqp1* transcripts were in epithelial cells (Figure 3, A and B, and Supplemental Figure 6). *Gpihbp1* ranked third in a list of genes expressed specifically in ChP ECs (relative to all other ChP cell types) (Supplemental Table 1). In scRNA-seq data from the mouse cerebral cortex (36), *Pecam1* transcripts were in ECs of the cerebral cortex, but *Gpihbp1* and *Plvap* transcripts were absent (Supplemental Figure 7).

**Figure 4:**
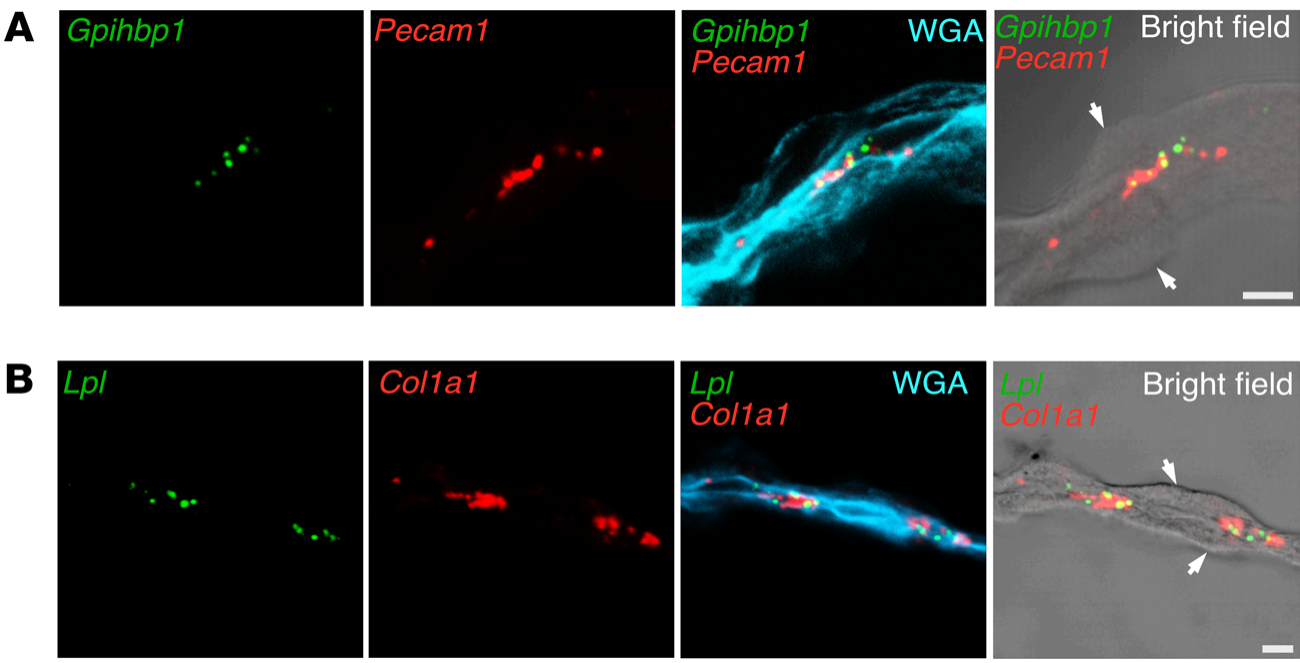
ISH studies, with RNAscope probes, of *Lpl*, *Col1a1*, *Gpihbp1*, and *Pecam1* expression in the ChP. ChP sections were stained with Alexa Fluor 488–wheat germ agglutinin (WGA). In addition to recording fluorescent signals from RNAscope probes, bright-field images were obtained. (**A**) ISH studies revealing *Gpihbp1* and *Pecam1* transcripts in ECs of ChP capillaries. (**B**) ISH studies revealing *Lpl* and *Col1a1* transcripts in ChP mesenchymal fibroblasts, which are adjacent to capillary ECs. White arrows in the bright-field images point to ChP epithelial cells. Because WGA was used to stain tissue sections (rather than being injected IV into mice), the WGA bound to HSPGs on both ChP capillaries and ChP epithelial cells. Images were recorded with a 63 × objective. Scale bar: 5 μm.

We also examined *Gpihbp1*, *Pecam1*, *Lpl*, and *Col1a1* expression in the mouse ChP by in situ hybridization. Consistent with the scRNA-seq findings (Figure 3, A and B, and Supplemental Figure 6), we observed overlap of *Gpihbp1* and *Pecam1* signals in ECs and overlap of *Lpl* and *Col1a1* expression in mesenchymal fibroblasts (located adjacent to capillary ECs [refs. 35, 37]) (Figure 4).

### TRLs marginate along ChP capillaries.

**Figure 5:**
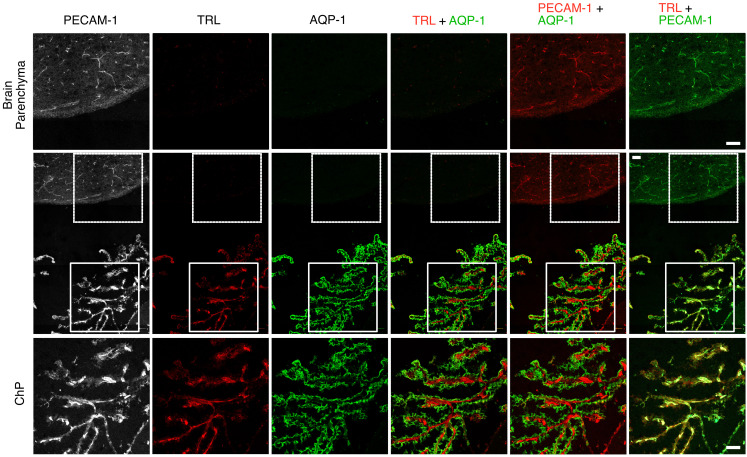
Confocal micrographs showing margination of TRLs along the luminal surface of ChP capillaries in WT mice. Alexa Fluor 488–2H8 (against PECAM-1; white) and Alexa Fluor 647–TRLs (red) were injected into the carotid artery. After 2 min, the vasculature was perfused with PBS and PFA. To identify ChP epithelial cells, sections were stained with an AQP-1–specific mAb followed by Alexa Fluor 555–anti-rabbit IgG (H+L) (green). Images were recorded with a 20 × objective. Scale bar: 50 μm. Boxed areas of the brain parenchyma and ChP are shown at higher magnification above and below, respectively.

An earlier study concluded that the margination of TRLs along heart capillaries depends on interactions with LPL (5). Consistent with that study, we observed robust margination of Alexa Fluor–labeled TRLs in heart capillaries of WT mice (Supplemental Figure 8). In heart capillaries of *Gpihbp1*^–/–^ mice, where LPL is absent (3), TRL margination along capillaries was absent (Supplemental Figure 8). Because our studies had documented LPL on the luminal surface of ChP capillaries, we suspected that we would observe TRL margination along ChP capillaries. To test that suspicion, we gave WT mice an IV injection of mAb 2H8 and TRLs (each labeled with an Alexa Fluor dye). After 2 minutes, the vasculature was perfused; sections were stained with an AQP-1–specific mAb; and microscopy was performed. TRL margination was observed in ChP capillaries (surrounded by AQP-1–positive ChP epithelial cells), but TRL margination was absent in capillaries of the adjacent brain parenchyma. PECAM-1 (detected by mAb 2H8) was in capillary ECs of the ChP and brain parenchyma (Figure 5). In independent experiments, WT mice were given an IV injection of 2H8, TRLs, and nonimmune goat IgG (each labeled with an Alexa Fluor dye). TRL margination was observed in PECAM-1–positive ChP capillaries; however, goat IgG was absent from capillaries, demonstrating that the perfusion of the vasculature had been effective in removing unbound antibodies (Supplemental Figure 9). As an additional test of vascular perfusion, WT mice were given an IV injection of TRLs and mAb 5D2 (which binds to human LPL but not mouse LPL). TRLs marginated along capillaries of the ChP, but mAb 5D2 was absent (demonstrating that perfusion of the vasculature removed unbound 5D2) (Supplemental Figure 10).

**Figure 6:**
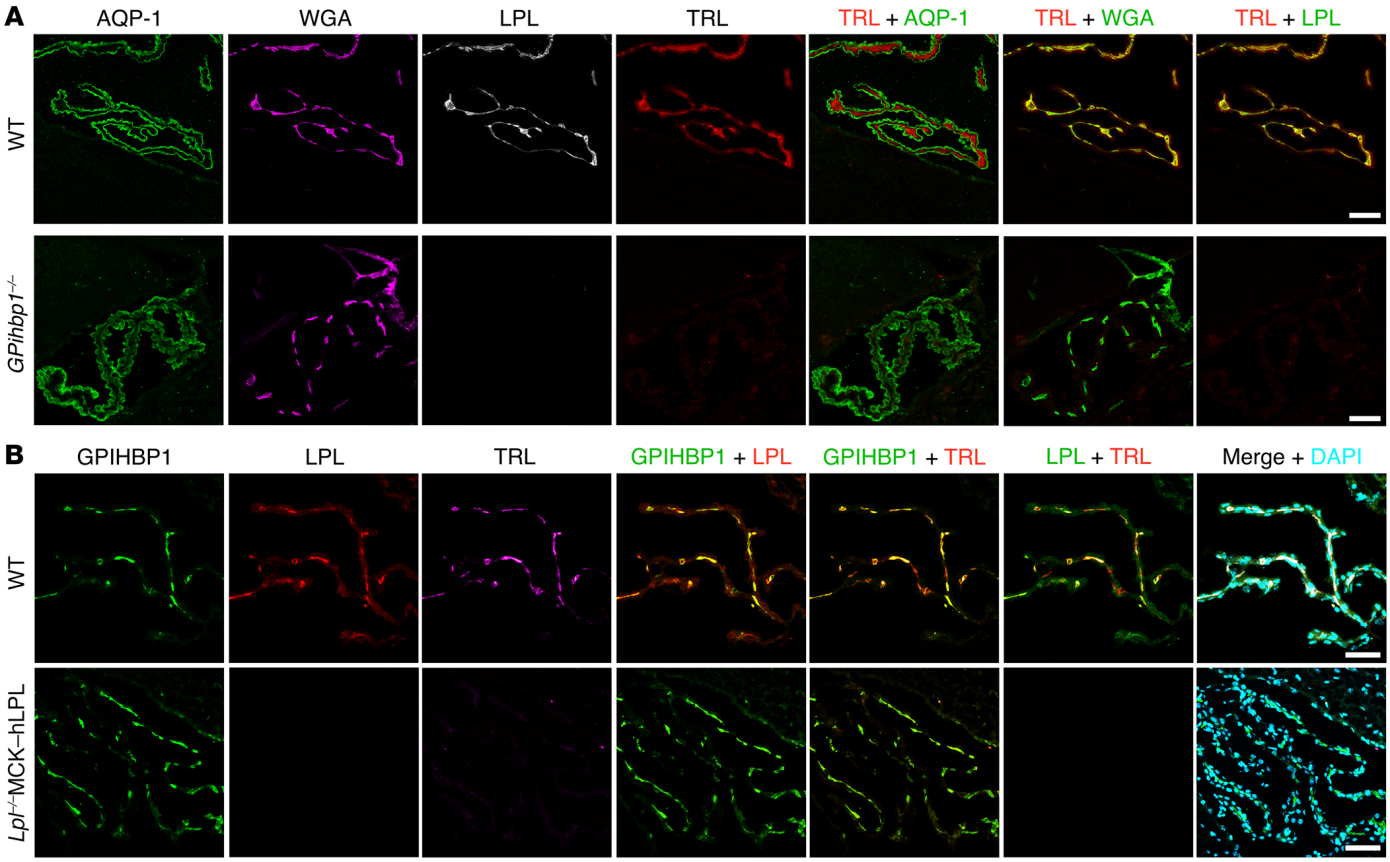
Confocal microscopy to assess TRL margination along ChP capillaries. (**A**) Assessing TRL margination in capillaries of *Gpihbp1^–/–^* mice and littermate WT mice. Alexa Fluor 555–WGA (purple), Alexa Fluor 488–Ab3175 (against mouse LPL, white), and Alexa Fluor 647–TRLs (red) were injected into the carotid artery of WT and *Gpihbp1^–/–^* mice. After 2 min, the vasculature was perfused extensively with PBS followed by PFA, and tissue sections were prepared for microscopy. ChP sections were stained with an AQP-1–specific mAb followed by Alexa Fluor Plus 405–anti-rabbit IgG (H+L) (green). Scale bar: 50 μm. (**B**) Assessing TRL margination in ChP capillaries of WT and *Lpl^–/–^*MCK–hLPL mice. Alexa Fluor 488–11A12 (against GPIHBP1, green), Alexa Fluor 555–Ab3175 (red), and Alexa Fluor 647–TRLs (purple) were injected into the carotid artery of WT and *Lpl^–/–^*MCK–hLPL mice. After 2 min, the vasculature was perfused with PBS followed by PFA, and sections were prepared for microscopy. Images were recorded with a 20 × objective. Scale bar: 40 μm.

We suspected that TRL margination would be absent in ChP capillaries of *Gpihbp1^–/–^* mice (where LPL is absent). To test this suspicion, WT and *Gpihbp1^–/–^* mice were given an IV injection of TRLs, WGA, and the mouse LPL–specific antibody Ab3175 (each labeled with an Alexa Fluor dye). After 2 minutes, the vasculature was perfused with PBS and sections were prepared for microscopy. In WT mice, we observed TRL margination and Ab3175 binding in WGA-stained ChP capillaries (Figure 6A). In *Gpihbp1^–/–^* mice, TRL margination and Ab3175 binding were absent in WGA-stained ChP capillaries (Figure 6A). We suspected that TRL margination would also be absent in ChP capillaries of *Lpl^–/–^*MCK–hLPL mice (where LPL is absent). WT and *Lpl^–/–^*MCK–hLPL mice were given an IV injection of TRLs, mAb 11A12, and Ab3175 (each labeled with an Alexa Fluor dye). 11A12 bound to GPIHBP1 in ChP capillaries of WT and *Lpl^–/–^*MCK–hLPL mice; Ab3175 binding and TRL margination were observed in ChP capillaries of WT mice but not *Lpl^–/–^*MCK–hLPL mice (Figure 6B).

### TRL margination along ChP capillaries was confirmed by electron microscopy.

**Figure 7:**
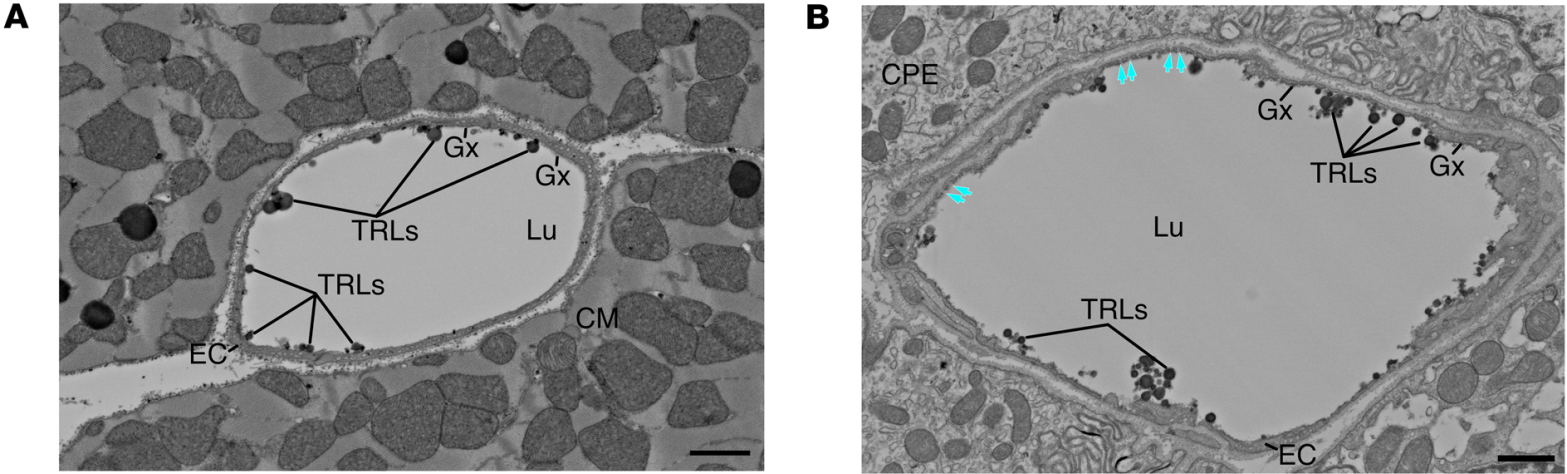
Scanning electron micrographs of thick sections from mouse heart (A) and mouse choroid plexus (B). The sections had been placed on a silicon wafer. These micrographs reveal margination of triglyceride-rich lipoproteins (TRLs) along the luminal surface of capillary endothelial cells (after giving WT mice an IV injection of TRLs). In Panel B, teal arrows point to endothelial cell fenestrations in a choroid plexus capillary. The EC glycocalyx was stained with LaCl_3_/DyCl_3_. Lu, lumen; Gx, glycocalyx; EC, endothelial cell; CM, cardiomyocyte. Scale bars: 1 μm.

In earlier studies (5, 7), we documented that LPL, including LPL within the glycocalyx, mediates margination of TRLs along capillaries. Here, we used electron microscopy to visualize TRL margination in heart and ChP capillaries. Following an IV infusion of TRLs and after extensive perfusion of the vasculature, we observed TRL margination in heart capillaries (including on the EC glycocalyx) (Figure 7A and Supplemental Figure 11). TRL margination was also observed in ChP capillaries (Figure 7B, Supplemental Figure 12, and Supplemental Figure 13). As expected, electron micrographs revealed fenestrations in ChP ECs (Figure 7B and Supplemental Figure 13) and a “forest” of microvilli at the apical surface of ChP epithelial cells (Supplemental Figure 13).

### Margination and processing of [^2^H]TRLs in ChP capillaries.

**Figure 8:**
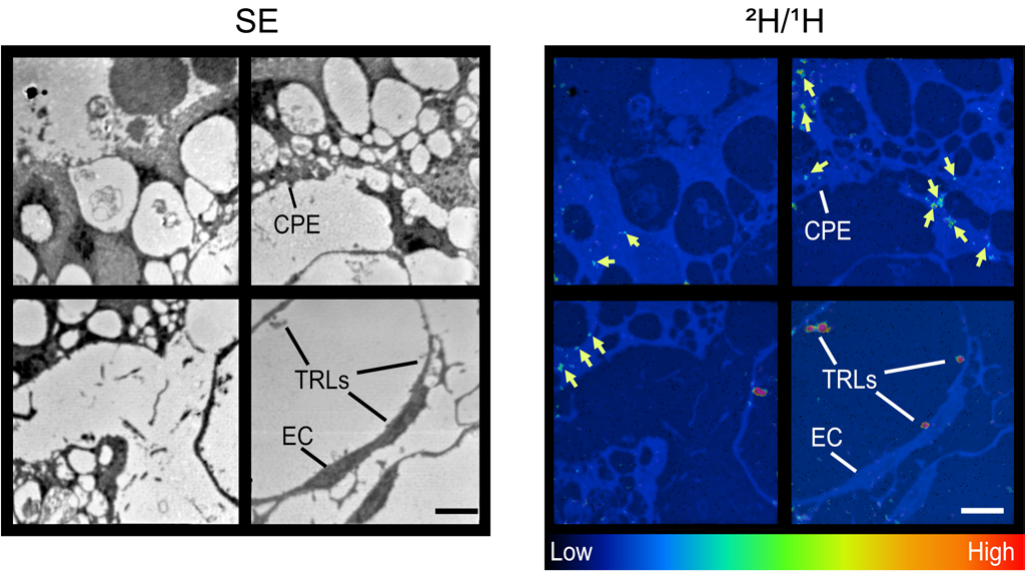
NanoSIMS images showing margination of [^2^H]TRLs along a ChP capillary and foci of ^2^H enrichment in ChP epithelial cells. Mice were given an IV injection of [^2^H]TRLs. After 5 min, the vasculature was perfused with a HEPES buffer followed by perfusion/fixation with the same buffer containing 2.5% (vol/vol) glutaraldehyde. Resin-embedded sections were prepared for NanoSIMS analyses. A secondary electron (SE) image, along with a ^2^H/^1^H ratio image, revealing [^2^H]TRL margination along the luminal surface of a ChP capillary and foci of ^2^H enrichment (yellow arrows) in ChP epithelial cells. EC, endothelial cell; CPE, ChP epithelial cells. Scale bar: 50 μm. The ^2^H/^1^H ratio in the TRLs was 0.000226 ± 0.000007 (mean ± SD) (*n* = 351); the ^2^H/^1^H ratio in the foci of ^2^H enrichment in ChP epithelial cells was 0.000171 ± 0.0000026 (*n* = 1387); the ^2^H/^1^H abundance in resin was 0.000120.

We isolated [^2^H]TRLs from the plasma of *Gpihbp1*^–/–^ mice that had been given uniformly-labeled [^2^H]fatty acids by gastric gavage for 4.5 days. We then gave WT mice an IV infusion of [^2^H]TRLs. After 5 minutes, the vasculature was perfused, and resin-embedded sections of the ChP were prepared for NanoSIMS analyses. ^2^H/^1^H NanoSIMS images revealed margination of [^2^H]TRLs along ChP capillaries and ^2^H enrichment in adjacent ChP epithelial cells (Figure 8).

### GPIHBP1, intracapillary LPL, and TRL margination in circumventricular organs.

**Figure 9:**
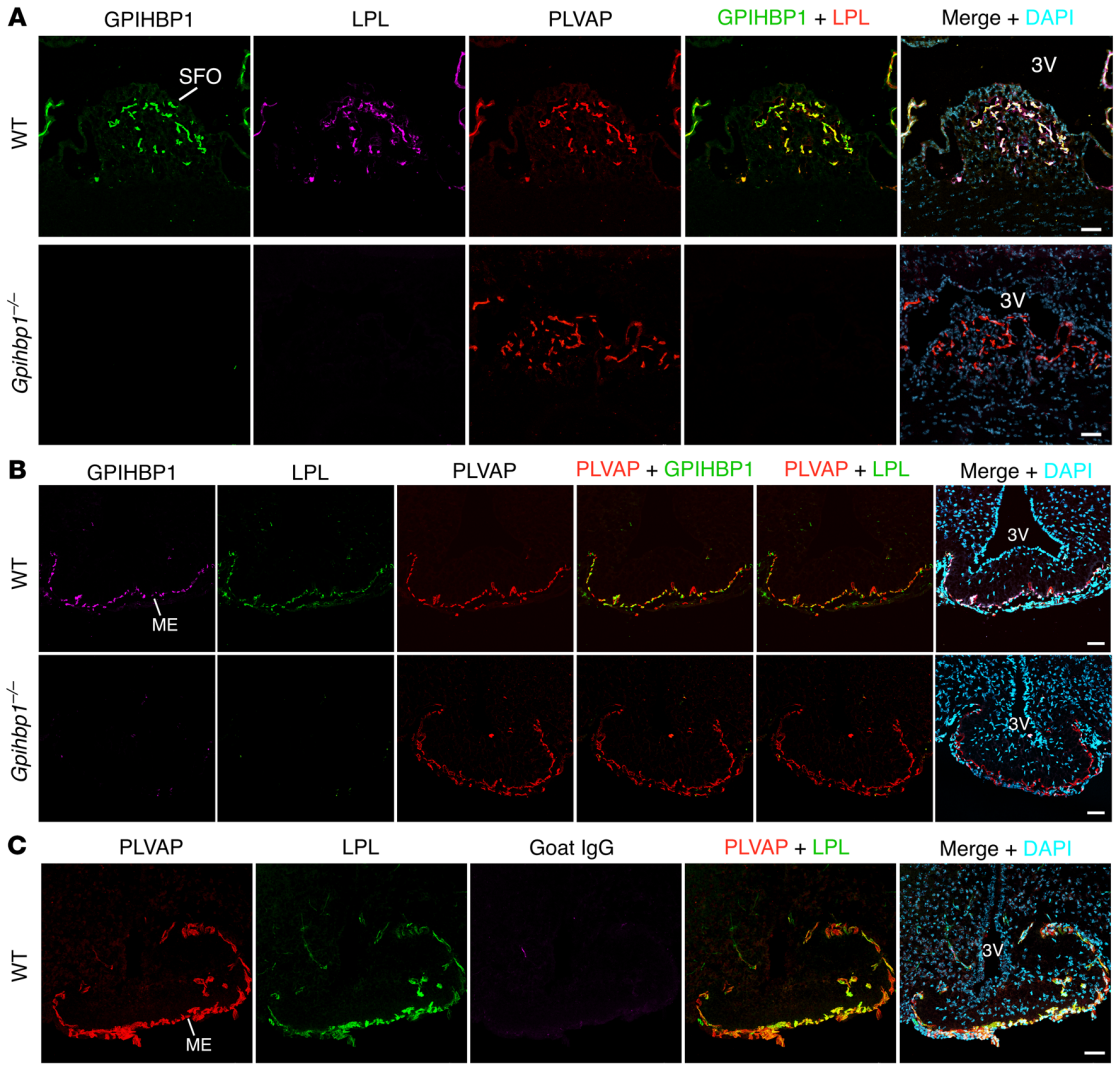
Confocal micrographs demonstrating GPIHBP1 and LPL on the luminal surface of SFO capillaries and median eminence (ME) capillaries in WT mice but not *Gpihbp1*^–/–^ mice. *Gpihbp1*^–/–^ mice and littermate WT mice were given an IV injection of Alexa Fluor 555–11A12 (against GPIHBP1), Alexa Fluor 647–27A7 (against mouse LPL), and Alexa Fluor 488–MECA-32 (against PLVAP). After 2 min, the vasculature was perfused with PBS and PFA, and tissue sections were prepared for microscopy. In **A**, GPIHBP1 is green; LPL is purple, and PLVAP is red. In **B**, GPIHBP1 is purple, LPL is green, and PLVAP is red. Shown in **A** are representative images from SFO capillaries (*n* = 3 independent experiments). Shown in **B** are representative images from ME capillaries (*n* = 2 independent experiments). **C** shows a control experiment documenting effective vascular perfusion of the ME. In this experiment, mice were given an IV injection of Alexa Fluor 647–Ab27A7 (against LPL; green) and Alexa Fluor 555–nonimmune goat IgG (purple). After 2 min, the vasculature was perfused with PBS and PFA; tissue sections were stained with mAb MECA-32 followed by Alexa Fluor 488–anti-rat IgG (H+L) (red). The absence of goat IgG in the capillary lumen indicates that the vasculature had been effectively perfused. All images in this figure were recorded with a 20 × objective. Scale bars: 50 μm. 3V, third ventricle.

Circumventricular organs (CVOs) are midline structures adjacent to the third and fourth ventricles containing fenestrated capillaries. Two of the CVOs, the subfornical organ (SFO) and the median eminence (ME), have been implicated in hypothalamic regulation of food intake (29–33). We suspected that we might find GPIHBP1 and LPL in SFO and ME capillaries. To test that suspicion, we gave WT and *Gpihbp1*^–/–^ mice an IV injection of mAb 27A7 (against LPL), mAb 11A12 (against GPIHBP1), and mAb MECA-32 (against PLVAP), each labeled with an Alexa Fluor dye. After 2 minutes, the vasculature was perfused extensively with PBS, and tissue sections were prepared for microscopy. In WT mice, we detected binding of mAbs 27A7, 11A12, and MECA-32 to the luminal surface of SFO capillaries (Figure 9A). In *Gpihbp1*^–/–^ mice, we observed binding of mAb MECA-32 to SFO capillaries, but there was no binding of mAb 11A12 or 27A7 (Figure 9A). Consistent with the SFO findings, we observed binding of mAbs 27A7, 11A12, and MECA-32 to the luminal surface of ME capillaries of WT mice (Figure 9B). In ME capillaries of *Gpihbp1*^–/–^ mice, we observed binding of MECA-32, but there was no binding of 11A12 or 27A7 (Figure 9B). In a control experiment, we observed binding of mAbs 27A7 and MECA-32 to ME capillaries of WT mice (Figure 9C). In this experiment, we had given mice an IV injection of goat IgG. Following perfusion of the vasculature, there was no goat IgG in ME capillaries (indicating that the perfusion of the vasculature had removed unbound antibodies) (Figure 9C).

A subclustering reanalysis of scRNA-seq data from the ME and neighboring brain parenchyma (36) revealed *Gpihbp1* expression in ECs that expressed *Plvap* and *Esm1* (markers of fenestrated endothelium) (Supplemental Figure 14). ECs lacking *Plvap* and *Esm1* expression, likely from adjacent brain tissue, were negative for *Gpihbp1* expression (Supplemental Figure 14).

**Figure 10:**
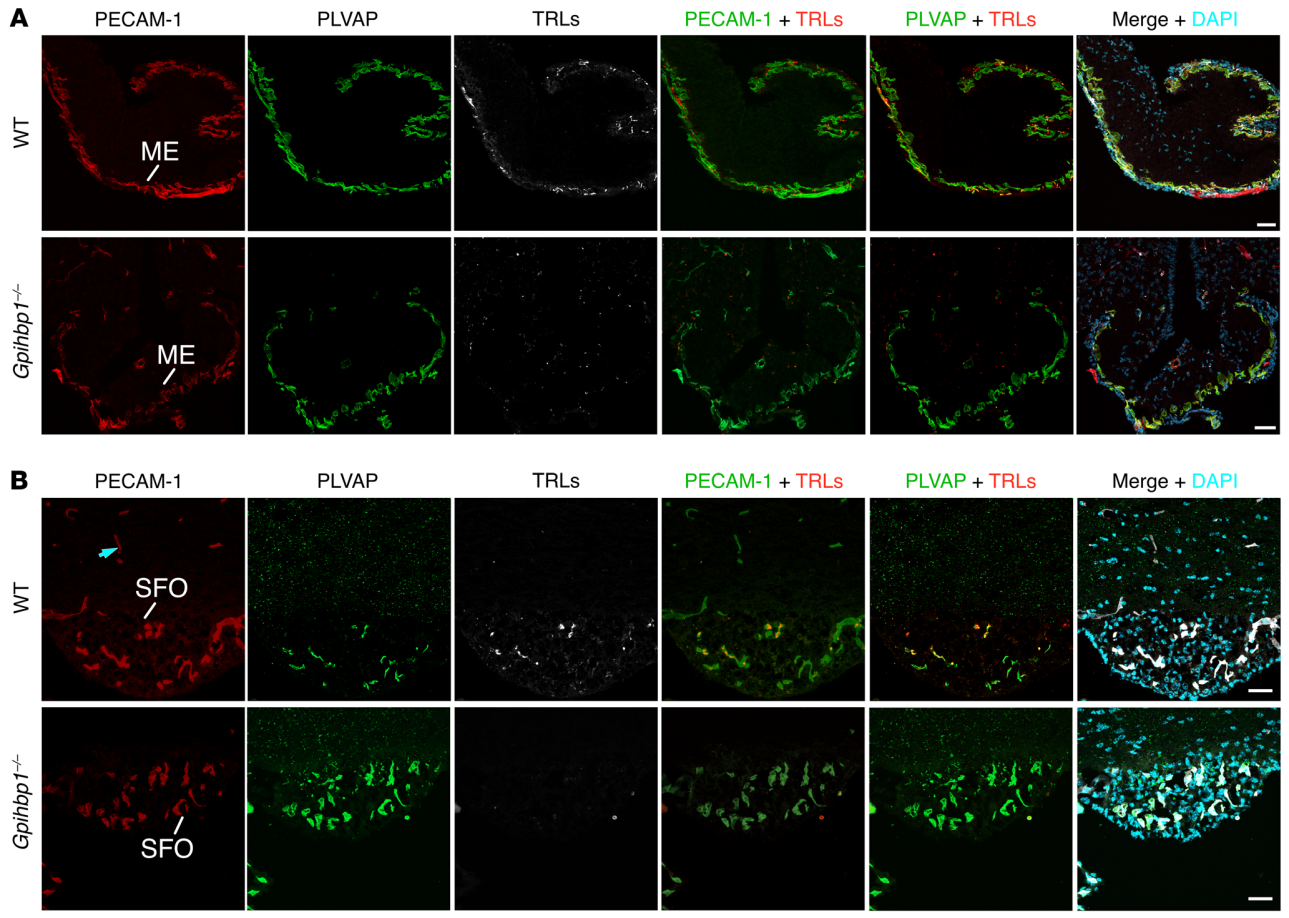
Margination of TRLs along the luminal surface of capillaries in mouse circumventricular organs. (**A**) TRL margination in capillaries of the median eminence (ME). The mice were given an IV injection of Alexa Fluor 647–TRLs, Alexa Fluor 488–mAb 2H8 (against PECAM-1), and Alexa Fluor 555–mAb MECA-32. After 2 min, the vasculature was perfused extensively, and tissue sections were prepared for confocal microscopy. TRL margination was observed in PLVAP1- and PECAM-1–positive ME capillaries in WT mice but not in *Gpihbp1^–/–^* mice. (**B**) TRL margination in capillaries of the subfornical organ (SFO). WT and *Gpihbp1^–/–^* mice were given an IV injection of Alexa Fluor 647–TRLs, Alexa Fluor 488–mAb 2H8 (against PECAM-1), and Alexa Fluor 555–mAb MECA-32. After 2 min, the vasculature was perfused extensively, and sections were prepared for microscopy. TRL margination was observed in PECAM-1– and PLVAP-positive SFO capillaries in WT mice but not in *Gpihbp1^–/–^* mice. Images were recorded with a 20 × objective. Scale bars: 50 μm. Panel B shows representative images from 3 independent experiments.

Because LPL was detected along the luminal surface of ME and SFO capillaries in WT mice, we suspected that TRLs would marginate along those capillaries. To test this possibility, WT and *Gpihbp1^–/–^* mice were given an IV injection of TRLs, mAb 2H8, and mAb MECA-32 (each labeled with an Alexa Fluor dye). TRL margination was observed in PLVAP1- and PECAM-1–positive ME capillaries of WT mice but not *Gpihbp1^–/–^* mice (Figure 10A). We also assessed TRL margination in SFO capillaries. WT and *Gpihbp1^–/–^* mice were given an IV injection of TRLs, 2H8, and mAb MECA-32 (each labeled with an Alexa Fluor dye). TRL margination was observed in PLVAP1- and PECAM-1–positive SFO capillaries in WT mice but not *Gpihbp1^–/–^* mice (Figure 10B).

### GPIHBP1 and LPL are expressed in human ChP.

**Figure 11:**
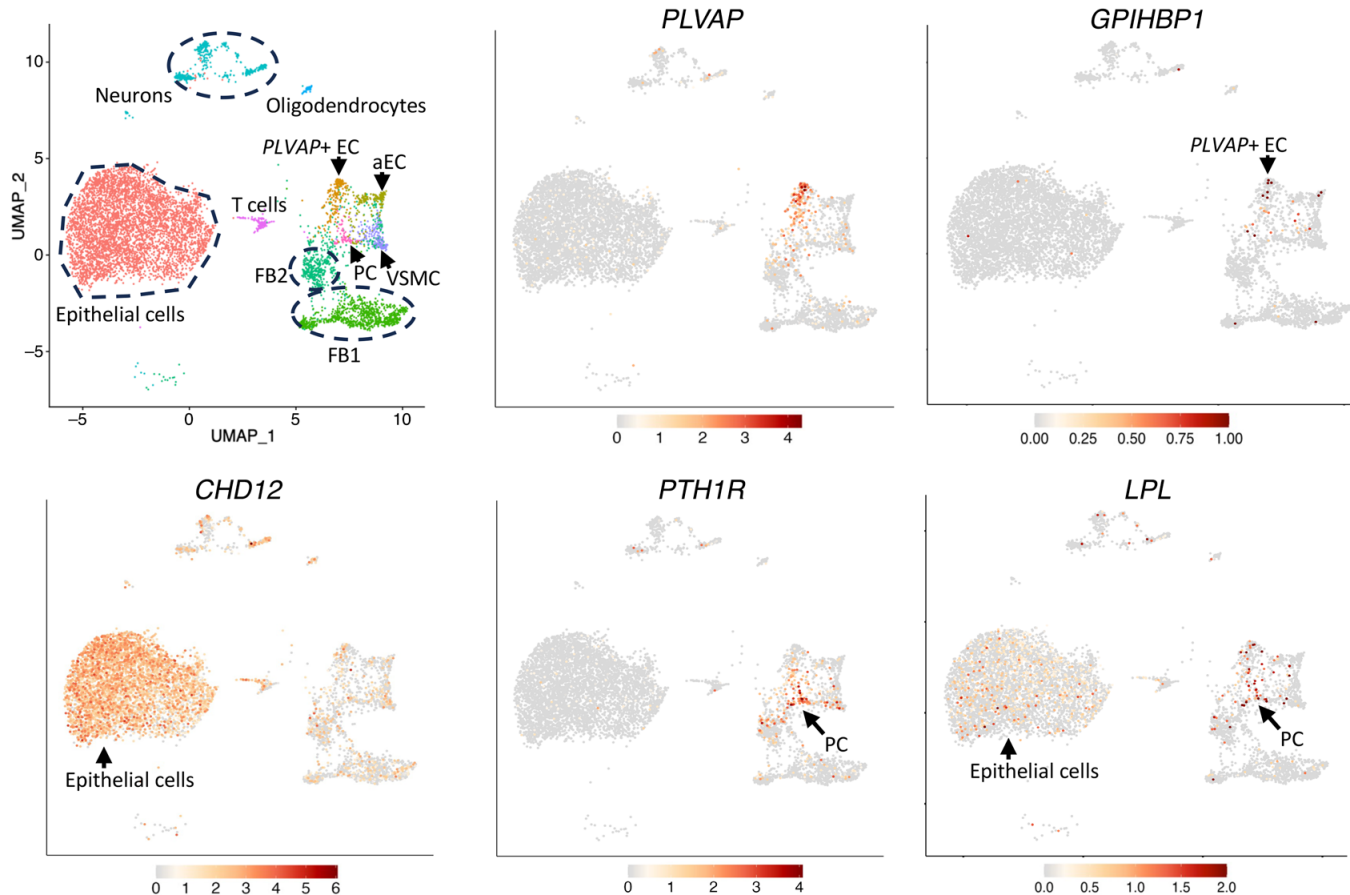
Transcriptomic analyses of disease-free human choroid plexus revealed that *GPIHBP1* is expressed in *PLVAP*-positive endothelial cells and that *LPL* is expressed by pericytes and epithelial cells. UMAPs of gene expression in epithelial cells, *PLVAP*+ ECs, arterial ECs (aEC), fibroblasts (FB1, FB2), neurons, oligodendrocytes, vascular smooth muscle cells (VSMC), T cells, and pericytes (PC). *GPIHBP1* transcripts were enriched in *PLVAP*+ ECs; *LPL* transcripts were enriched in pericytes (which express *PTH1R*) and epithelial cells (which express *CDH12*). These data were generated by re-analyzing a dataset (GEO: GSE264154) in a published study (38).

snRNA-seq studies of disease-free human ChP (38) revealed that *GPIHBP1* is expressed in ECs that are positive for *PLVAP* (a marker of fenestrated ECs) and that *LPL* is expressed in pericytes and epithelial cells (Figure 11 and Supplemental Figures 15 and 16). The expression of *GPIHBP1* in *PLVAP*-positive ECs (*PLVAP*+) was higher than in all other cell types (*P* = 2.80 × 10^–40^) (Supplemental Figure 15). *LPL* expression was higher in pericytes (*P* = 2.37 × 10^–20^) and ChP epithelial cells (*P* = 4.85 × 10^–49^) than in other ChP cell types (Supplemental Figure 15). Initially, it appeared that *LPL* might also be expressed in *PLVAP*+ ECs (Supplemental Figure 15), but that was almost certainly due to pericyte contamination (Supplemental Figure 16, A and B). Among 182 *PLVAP*+ ECs, 29 were *LPL*+ and 153 were *LPL*–; the *LPL*+ ECs were enriched in pericyte markers, whereas the *LPL*– cells were not (Supplemental Figure 16B).

**Figure 12:**
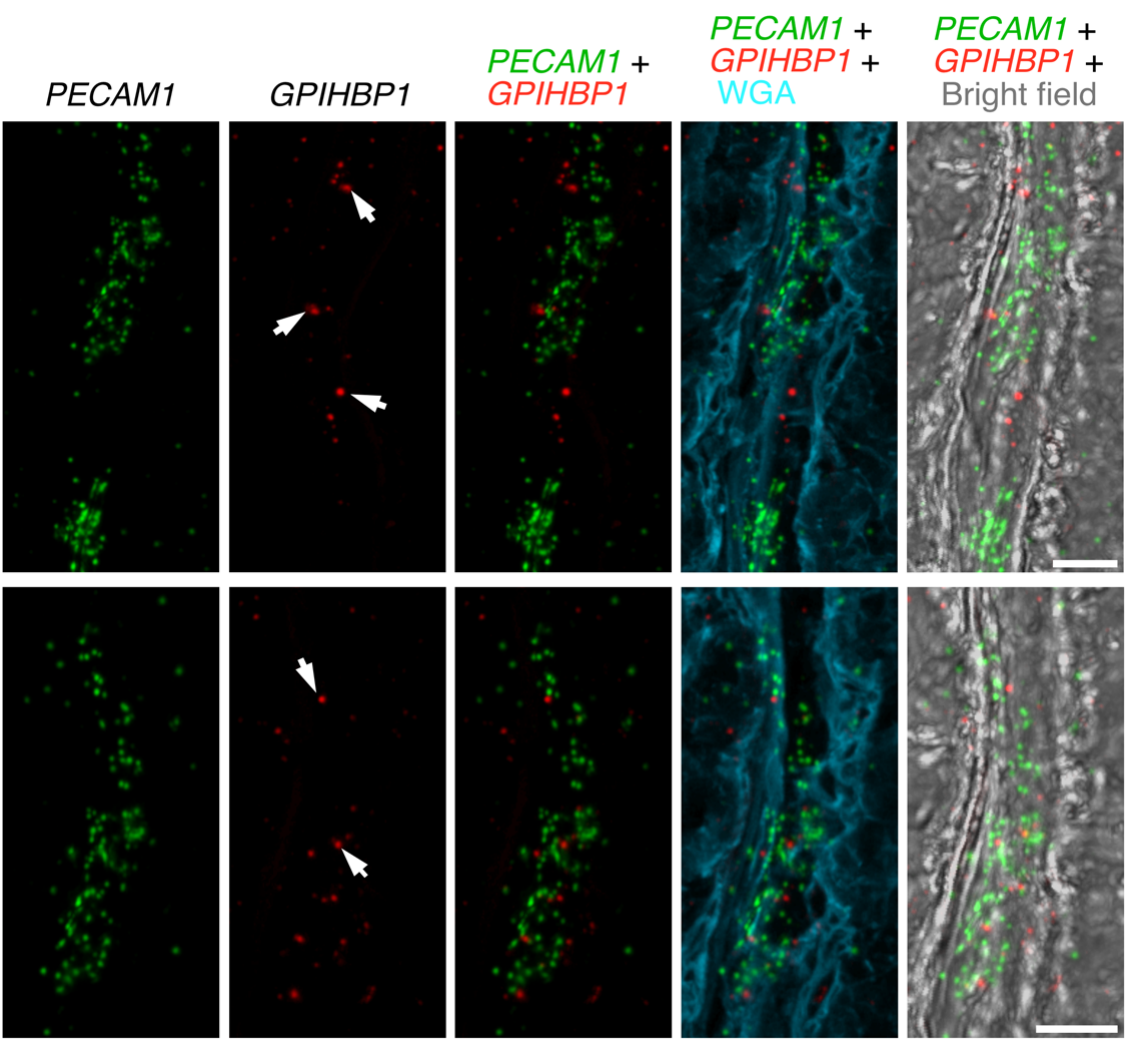
ISH studies — with RNAscope probes — of *PECAM1* expression and *GPIHBP1* expression in human ChP. Sections were stained with Alexa Fluor 488–wheat germ agglutinin (WGA). *PECAM1* transcripts (green) were recorded in the 647 channel; *GPIHBP1* transcripts (red) were recorded in the 568 channel. WGA and bright-field images were helpful for defining ChP morphology. Images were recorded with a 20 × objective. Scale bar: 10 μm.

**Figure 13:**
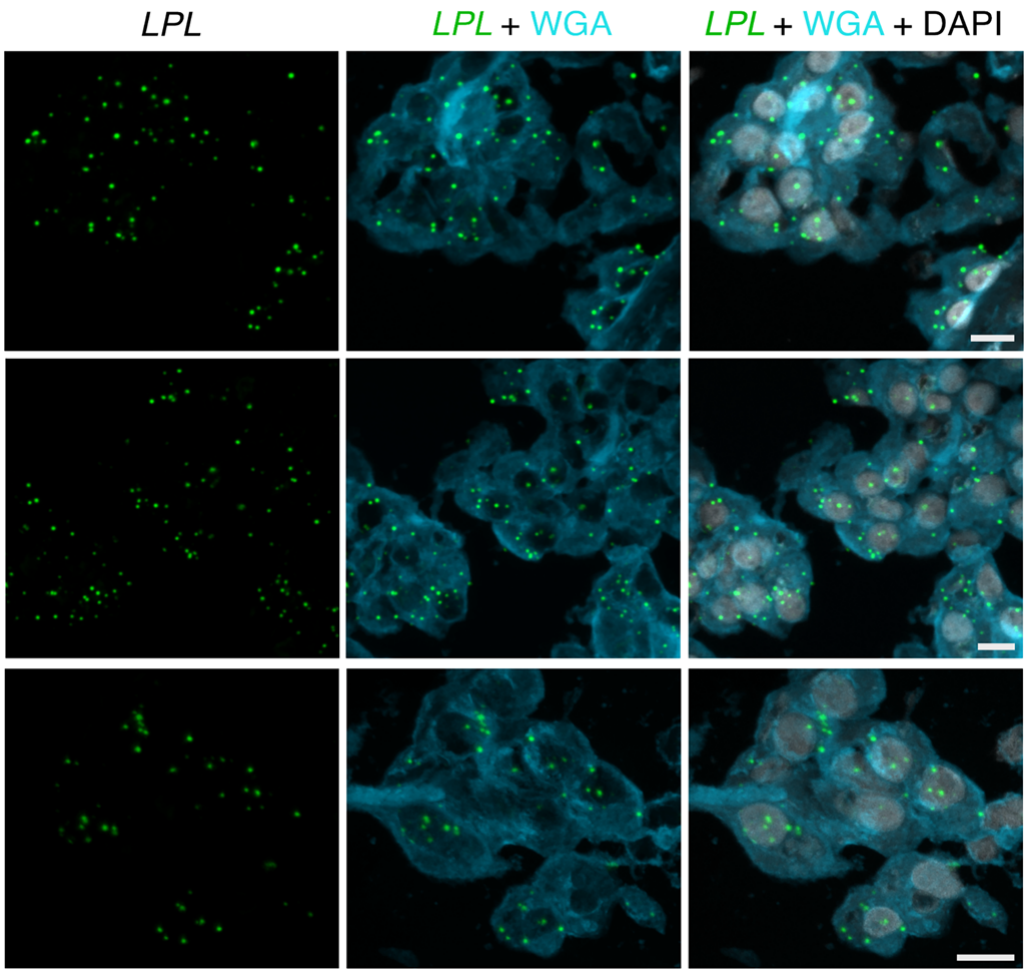
ISH studies — with RNAscope probes — of *LPL* transcripts in epithelial cells of the human ChP. Sections were stained with Alexa Fluor 488–wheat germ agglutinin (WGA). *LPL* transcripts (green) were recorded in the 647 channel. WGA was helpful for visualizing ChP epithelial cell morphology. The top 2 images were recorded with a 20 × objective; the bottom image was recorded with a 63 × objective. Scale bar: 10 μm.

**Figure 14:**
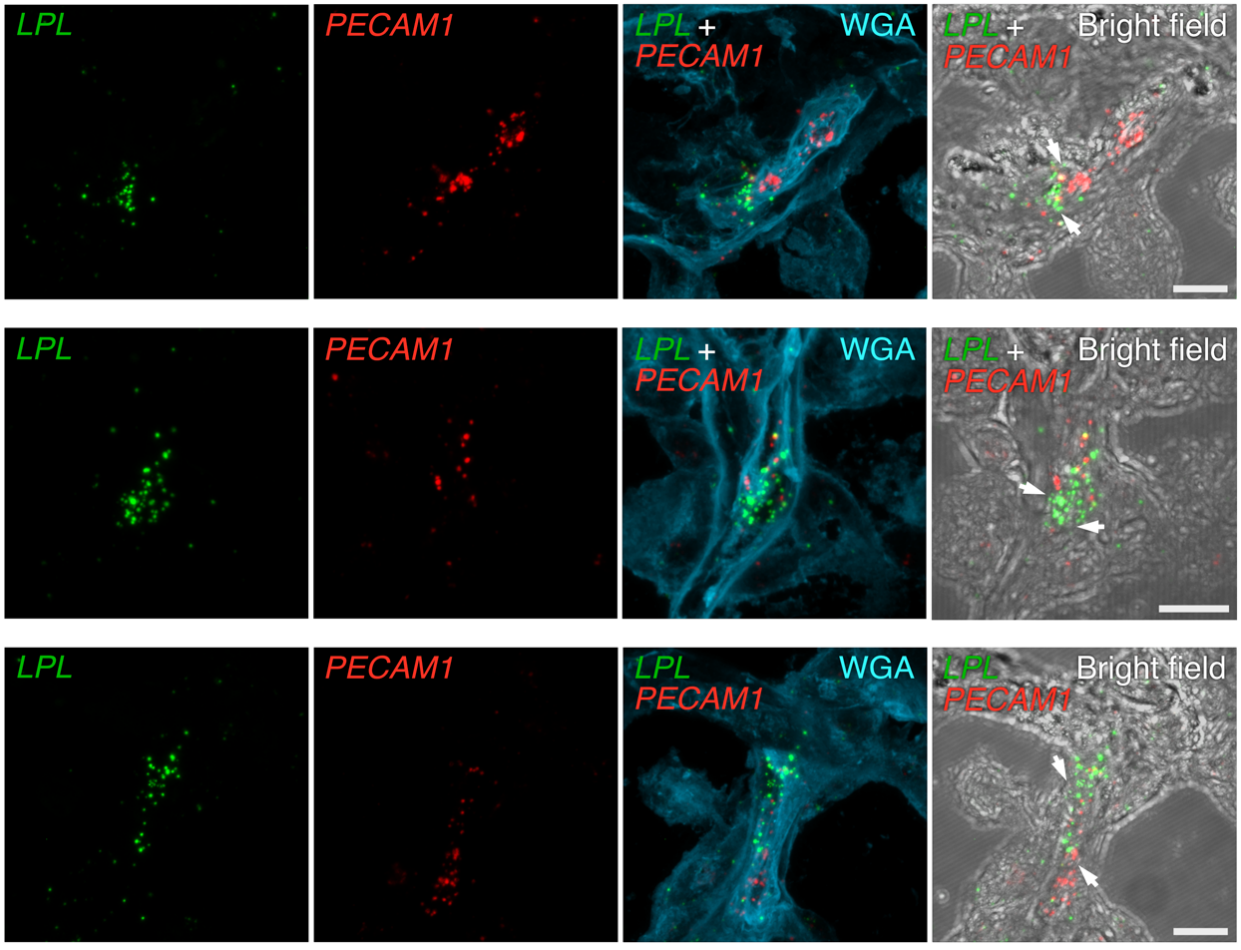
ISH studies — with RNAscope probes — of *LPL* and *PECAM1* expression in human ChP. Sections were stained with Alexa Fluor 488–wheat germ agglutinin (WGA). *LPL* transcripts (green) were recorded in the 568 channel; *PECAM1* transcripts (red) were recorded in the 647 channel. WGA and bright-field images were helpful for delineating ChP morphology. Transcriptomic studies revealed that *LPL* is expressed in pericytes and epithelial cells of the human ChP. The overlap of *LPL* transcripts (green) and *PECAM1* transcripts (red) is consistent with *LPL* expression in pericytes and *PECAM1* expression in ECs. Images were recorded with a 63 × objective. Scale bar: 10 μm.

In situ hybridization studies of human ChP were consistent with the transcriptomics studies. We observed overlap of *GPIHBP1* and *PECAM1* transcripts in ChP ECs (Figure 12). We observed *LPL* transcripts in ChP epithelial cells, which had been stained by WGA (Figure 13). We also observed close spatial proximity of *LPL* and *PECAM1* transcripts in ChP capillaries (Figure 14), consistent with the expression of *PECAM1* in ECs and *LPL* expression in adjacent pericytes.

We used qRT-PCR to assess *GPIHBP1*, *LPL*, and *PECAM1* expression, relative to *GAPDH*, in human ChP and heart (Supplemental Figure 17). *GPIHBP1* and *PECAM1* were expressed in roughly similar amounts in ChP and heart. Not surprisingly, *LPL* transcripts were much higher in the heart than in the ChP (Supplemental Figure 17). The *GPIHBP1/LPL* expression ratio was approximately 9.2 fold higher in ChP than in the heart (Supplemental Figure 17).

## Discussion

In earlier studies, we found no evidence for GPIHBP1, intracapillary LPL, or TRL margination in BBB capillaries of the mouse cerebral cortex or cerebellum (5, 7, 16). However, because free fatty acids had been reported to play signaling roles in the rodent brain (18, 19), we were reluctant to dismiss the possibility of intravascular TRL processing in the CNS. In the current studies, we observed GPIHBP1 (LPL’s dedicated endothelial cell transporter) on the luminal surface of capillaries in the mouse ChP. We also detected LPL along the luminal surface of ChP capillaries in WT mice (but not *Gpihbp1^–/–^* mice). Those findings indicated that GPIHBP1-mediated LPL transport is required for the presence of LPL along the luminal surface of ChP capillaries. We also observed TRL margination along the luminal surface of ChP capillaries in WT mice (where LPL is present) but not in ChP capillaries of *Gpihbp1^–/–^* and *Lpl^–/–^*MCK-hLPL mice (where LPL is absent). After giving mice an IV injection of [^2^H]TRLs, we observed, by NanoSIMS imaging, [^2^H]TRL margination along ChP capillaries along with ^2^H enrichment in adjacent ChP epithelial cells. The expression of GPIHBP1 and LPL in the ChP was not a peculiarity of mice. Transcriptomic studies of disease-free human ChP (38) revealed *GPIHBP1* transcripts in capillary ECs and *LPL* transcripts in pericytes and epithelial cells. Those findings were strongly supported by in situ hybridization studies and RT-PCR studies on human ChP.

Intravascular lipolysis in the mouse CNS was not confined to the ChP. We also observed GPIHBP1 expression, intracapillary LPL, and TRL margination in capillaries of the SFO and ME, which are circumventricular organs that have been implicated in the hypothalamic regulation of food intake (29–33).

As noted in the Introduction, an earlier study reported that a deficiency of LPL in neurons was associated with obesity in mice (23). In that study, TRL processing was investigated in the CNS of WT and neuron-specific *Lpl* knockout mice. Both groups of mice were given an IV injection of [^3^H]TRLs, and after 15 minutes, tritium counts in the hypothalamus, hippocampus, hindbrain, and forebrain were quantified. The amounts of tritium in the hypothalamus were slightly greater in WT mice than in neuron-specific *Lpl*-knockout mice (*P* = 0.047), but amounts of tritium in the hippocampus, hindbrain, and forebrain were slightly lower in the WT mice. Whether the tritium in the brain resulted from [^3^H]free fatty acids in the [^3^H]TRL preparation, LPL-mediated release of [^3^H]fatty acids in peripheral tissues, or LPL-mediated [^3^H]fatty acid release within the CNS was not clear. In considering these findings, the authors offered a model showing TRLs interacting with HSPG-bound LPL inside a BBB capillary and proposed that the LPL in BBB capillaries mediates entry of TRL remnants and TRL-derived lipids into the brain parenchyma (39). In this model, it was unclear whether the LPL inside BBB capillaries originated from neurons, and, if so, how the neuronal LPL would have reached the luminal surface of capillaries. Earlier studies had already established that GPIHBP1, the protein that shuttles LPL into capillaries, is absent in BBB capillaries in the mouse brain parenchyma (3, 40, 41).

Our current studies were not designed to explore the function of neuronal LPL in the mouse brain. Rather, our goal was to investigate the possibility of TRL processing in specialized vascular beds within the CNS. We documented GPIHBP1, LPL, and TRL margination in fenestrated capillaries of the ChP, SFO, and ME of WT mice (but not *Gpihbp1^–/–^* mice). The existence of the machinery for intravascular lipolysis in the mouse CNS suggested a potential mechanism by which lipoproteins in the systemic circulation could contribute to fatty acid signaling within the CNS.

The levels of *Gpihbp1* and *Lpl* transcripts in the mouse ChP, relative to *Pecam1* transcripts, were less than 10% of levels in the mouse heart. Because the ChP is a small structure and because the levels of *Gpihbp1* and *Lpl* transcripts in the ChP were lower than in the heart, it seems highly unlikely that TRL processing in the ChP would contribute in a meaningful way to the energy demands of the brain parenchyma. Instead, we suspect that TRL processing in the ChP exists primarily to support the energy demands of transporter proteins in ChP epithelial cells, which are essential for maintaining ionic barriers and for producing cerebrospinal fluid (42). ChP epithelial cells contain abundant mitochondria ([12%–15%] of the volume of the cell [ref. 42]), and we suspect that the fatty acids from TRL processing in the ChP are mainly used to produce ATP in ChP epithelial cells. However, because our earlier NanoSIMS studies on the mouse heart had shown that TRL-derived fatty acids move very rapidly across ECs and into cardiomyocytes (11), we believe that it is possible, perhaps even likely, that some of the fatty acids derived from TRL processing in the ChP escape into the cerebrospinal fluid (43). Similarly, we suspect that some of the fatty acids from TRL processing in the circumventricular organs could reach the cerebrospinal fluid as well as neurons in adjacent anatomical structures (e.g., hypothalamus).

The absence of intracapillary LPL and TRL margination in the ChP of *Gpihbp1*^–/–^ mice was not accompanied by histopathology. That was not surprising. The absence of intracapillary LPL and TRL processing in the heart of *Gpihbp1*^–/–^ mice is not associated with heart failure or histopathology (3, 11, 41), very likely because the heart is capable of utilizing the free fatty acids and glucose for fuel (44). Similarly, we have not observed abnormalities in body weight or adiposity in *Gpihbp1*^–/–^ mice (45, 46), likely because adipocytes can produce triglycerides by de novo lipogenesis (47, 48). The absence of a body weight abnormality in adult *Gpihbp1*^–/–^ mice was not surprising. In adult *Lpl*^–/–^ mice (rescued from embryonic lethality with an LPL adenovirus), body weights were identical to the weights of WT mice, and there was no obvious organ pathology (49).

We found evidence, by single nucleus RNA sequencing (snRNA-seq) analyses, in situ hybridization studies, and qRT-PCR studies, for *GPIHBP1* and *LPL* expression in the human ChP. GPIHBP1 in the human ChP is expressed in PLVAP-positive ECs, whereas LPL is produced by pericytes and epithelial cells. The LPL secreted those cells is destined to be captured by GPIHBP1 and transported to the capillary lumen. We suspect that GPIHBP1 and LPL are present in fenestrated capillaries of centriventricular organs in humans (as they are in the mouse), but that possibility has not yet been investigated.

The ChP is present in all vertebrate species, but it is notable that lower vertebrates (e.g., birds, fish) do not express GPIHBP1 (2, 50) (3). In chicken and zebrafish heart, LPL is expressed by capillary ECs (2) rather than by cardiomyocytes, thereby obviating a requirement for GPIHBP1-mediated transport of LPL. Given those observations, it would not be surprising if LPL in the ChP of lower vertebrates were produced directly by capillary ECs.

## Methods

### Sex as a biological variable.

Our studies examined both male and female animals, and virtually identical findings were obtained in both male and female mice.

### Mice.

C57BL/6J mice were purchased from The Jackson Laboratory. Our studies were performed in male and female C57BL/6J mice at 8–12 weeks of age. Mice were fed a chow diet and were housed in a barrier facility with a 12-hour light–dark cycle. *Gpihbp1*^–/–^ and *Lpl*^–/–^MCK-hLPL mice (*Lpl*^–/–^ mice expressing a human LPL transgene driven by the muscle creatine kinase promoter) have been described previously (41, 51). All studies were approved by UCLA’s Animal Research Committee.

### Human tissues.

Frozen human samples and freshly isolated choroid plexus samples were obtained from UCLA’s Translational Pathology Core Laboratory, a service dedicated to tissue procurement, storage, and provision. Choroid plexus was harvested by neuropathologists within 24 h after death.

### Antibodies.

Mouse LPL (mLPL) was detected with rat monoclonal antibody (mAb) 27A7 and rabbit polyclonal antibodies (Ab3174, Ab3175). 27A7 binds to LPL’s C-terminal domain of mLPL (52); Ab3174 and Ab3175 bind almost exclusively to the N-terminal domain of mLPL (4). GPIHBP1 was detected with rat mAb 11A12 (53); PECAM-1 was detected with hamster mAb 2H8 (Developmental Studies Hybridoma Bank, University of Iowa) (54) or with a polyclonal goat antibody (R&D Systems, AF3628); PLVAP was detected with rat mAb MECA-32 (from BD Biosciences and the Developmental Studies Hybridoma Bank); AQP-1 was detected with a rabbit monoclonal antibody (Thermo Fisher Scientific, PA5-120324). We used the following Alexa Fluor dye–labeled secondary antibodies: Donkey anti-rabbit IgG (H+L) highly cross-adsorbed secondary antibody, Alexa Fluor plus 405 (Thermo Fisher Scientific, A48258); Donkey anti-goat IgG (H+L) cross-adsorbed secondary antibody, Alexa Fluor 488 (Thermo Fisher Scientific, A-31556); Alexa Fluor 647 Affinipure goat anti-Rat IgG (H+L) (Jackson ImmunoResearch, 112-605-003); Donkey anti-rabbit IgG (H+L) highly cross-adsorbed secondary antibody, Alexa Fluor 555 (Thermo Fisher Scientific, A-31572); Donkey anti-rat IgG (H+L) highly cross-adsorbed secondary antibody, Alexa Fluor 488 (Thermo Fisher Scientific, A-21208).

### Immunohistochemical studies.

To detect GPIHBP1 and PECAM-1 in the choroid plexus, 10-μm-thick frozen sections of the mouse brain were prepared, fixed with methanol at –20°C for 10 min, and incubated in blocking buffer (PBS containing 0.2% BSA and 5% donkey serum) for 1 h at RT. Sections were incubated with antibodies against GPIHBP1 (mAb 11A12, 5 μg/mL) and PECAM-1 (the polyclonal goat antibody, 5.7 μg/mL) at 4°C overnight. Sections were washed 3 times to remove unbound antibodies and then incubated with secondary antibodies for 45 min (Alexa Fluor 488–anti-goat IgG, Alexa Fluor 647–anti-mouse IgG at 1:200 dilution). After washing, the sections were postfixed with 3% PFA for 5 min, and cell nuclei were stained with DAPI. Images were recorded with an LSM980 microscope (Zeiss) with a 20 × objective (Objective Plan-Apochromat 20/0.8 M27 lens from Zeiss) with a 495/517-nm and 653/668-nm excitation/emission filter.

### Assessing binding of Alexa Fluor–labeled mAbs to the luminal surface of capillaries.

To assess expression of LPL, GPIHBP1, and PECAM-1 on the luminal surface of capillaries, mice were given an IV injection of Alexa Fluor 647–Ab3174, Alexa Fluor 555–27A7, Alexa Fluor 488–2H8 (150–500 μg in 0.2 ml of PBS). To assess expression of LPL and GPIHBP1 in ChP capillaries, mice were given an IV injection of Alexa Fluor 647–27A7, Alexa Fluor 555–11A12, and Alexa Fluor 488–wheat germ agglutinin (WGA) (150–500 μg in 0.2 ml of PBS). To assess LPL expression in ME capillaries, mice were given an IV injection of Alexa Fluor 647–27A7 and Alexa Fluor 555–goat IgG (as an experimental control to assess the effective perfusion of the vasculature). To assess LPL and GPIHBP1 expression in ME and SFO capillaries, mice were given an IV injection of Alexa Fluor 488–MECA-32, Alexa Fluor 647–27A7, and Alexa Fluor 555–11A12. After 2 min, mice were perfused with PBS (20 ml) through the left ventricle and perfusion-fixed with 3% PFA (10 ml). Tissues were embedded in optimal cutting temperature (OCT) compound, and 10-μm-thick tissue sections were prepared for confocal microscopy. In some experiments, tissue sections were stained with an AQP-1–specific mAb (to identify ChP epithelial cells) or with mAb MECA-32 (against PVLAP), followed by Alexa Fluor dye–labeled secondary antibodies. Images were recorded with an LSM980 microscope (Zeiss) with a 20 × objective (Objective Plan-Apochromat 20/0.8 M27 lens from Zeiss with 353/465-nm, 493/517-nm, 577/603-nm, and 653/668-nm excitation/emission filters).

### Calculation of Pearson colocalization coefficient.

Colocalization between PECAM-1 (488 channel) and GPIHBP1 (647 channel) in Figure 1 was assessed with Pearson’s correlation coefficient. The Pearson’s correlation coefficient (PCC) is used to evaluate the colocalization because it quantifies the pixel-by-pixel covariance between the signal intensities of 2 images. By subtracting the mean intensity from each pixel value, PCC becomes independent of overall signal intensity and background offset (55). Calculations were performed with ZEN 3.5 software (Zeiss). We used Zen software to perform image analyses because it allows seamless pixel-by-pixel colocalization analysis directly on the original files, eliminating the need to analyze images exported into another imaging program. The Zen software also provides users with the flexibility to define regions of interest and apply thresholds. In our studies, we excluded irrelevant pixels by restricting analyses to pixels in which the intensities were above a threshold value, as recommended (55). In our studies, pixel resolution for analyzing colocalization in choroid plexus images was 0.233 μm (1376 × 1376 pixel images); the pixel resolution for analyzing colocalization in the heart was 0.122 μm (2024 × 2024 pixels).

### In situ hybridization studies.

In situ hybridization (ISH) experiments were performed with RNAscope probes and the RNAscope Multiplex Fluorescent Detection Kit v2.0 (ACD Bio Techne, CA, USA) (2). Paired double-Z oligonucleotide probes were prepared by ACD Bio Techne. 10-μm-thick cryosections were incubated in 3% PFA for 1.5 h followed by a 10-min incubation with H_2_O_2_ at room temperature. Protease III treatment was carried out for 30 min at room temperature, followed by probe hybridization for 2 h at 40°C in a HybEZ II Oven. Probe signals were detected with Tyramide signal amplification (TSA) vivid fluorophore 570/650. Slides were incubated with Alexa Fluor 488–WGA (20 μg/ml) at RT for 1 h and then stained with DAPI. Images were recorded with the LSM980 microscope with a 20 objective (Objective Plan-Apochromat 20/0.8 M27 lens from Zeiss) or a 63 objective (Objective Plan-Apochromat 63/1.4 Oil DIC M27 lens from Zeiss) with 353/465-nm, 493/517-nm, 548/561-nm, and 650/673-nm excitation/emission filters. Bright-field images were recorded with the transmitted light detector (T-PMT).

### Assessing margination of triglyceride-rich lipoproteins (TRLs) along the luminal surface of capillaries.

TRLs were isolated from the plasma of chow-fed *Gpihbp1*^–/–^ mice by ultracentrifugation (5). WT mice were injected IV with 100 μl of normal saline containing 5 μl of 51 mM tetrahydrolipstatin (THL). After 2 min, mice were given an IV injection of Alexa Fluor 647–TRLs (300 μg of TRL protein in 200–300 μl PBS), Alexa Fluor 555–2H8, and 5 μl of 51 mM THL. In other experiments, WT and *Lpl*^–/–^MCK–hLPL mice were given an intracarotid injection of Alexa Fluor 647–TRLs, Alexa Fluor 555–Ab3175, Alexa Fluor 488–11A12, and 100 μl normal saline containing 5 μl of 51 mM THL; *Gpihbp1*^–/–^ and WT mice were given an intracarotid injection of Alexa Fluor 647–TRLs, Alexa Fluor 555–WGA, Alexa Fluor 488–Ab3175, and 5 μl of 51 mM THL in 200 μl normal saline. To assess TRL margination in SFO capillaries, WT mice were given an IV injection of Alexa Fluor 488–TRLs, Alexa Fluor 647–2H8, Alexa Fluor 555–MECA-32, and 5 μl of 51 mM THL. To assess TRLs margination in ME capillaries, WT mice were given an IV injection of Alexa Fluor 647–TRLs, Alexa Fluor 488–2H8, Alexa Fluor 555–MECA-32, and 5 μl of 51 mM THL. After 2 min, mice were perfused with 20 ml of PBS (containing 10 mM EDTA) through the left ventricle and then perfusion-fixed with 3% PFA (10 ml). In some experiments, sections were stained with mAbs against AQP-1 or PVLAP, followed by Alexa Fluor dye–labeled secondary antibodies. Images were recorded with an LSM980 microscope (Zeiss) with a Plan-Apochromat 20/0.8 M27 lens from Zeiss).

### Single-cell transcriptomics.

A single-cell transcriptomic dataset for embryonic mouse ChP was retrieved from GEO: GSE168704 (35). In those studies, LV, 3V, and 4V ChP was micro-dissected and pooled; live cells were sorted by FACS (35); and single-cell sequencing was performed. For our analyses, we removed cells with mitochondrial gene content > 6%, cells with less than 1,000 or more than 6,000 genes, and cells with unique molecular identifier (UMI) counts greater than 30,000. Using Seurat (v5.0.3) in R (v4.3.0), counts were normalized with a scale factor of 10,000 reads/cell, and variable features were identified with the variance-stabilizing transformation (vst) method. Data were then scaled and reduced by principal component analysis (PCA). A shared nearest neighbor (SNN) graph was generated from the top 30 principal components (PCs) with the FindNeighbors function, which facilitated Louvain clustering with the FindClusters function. UMAP analyses were performed for visualization of different cell types.

Cell-type annotations were performed manually with marker genes. We used the Seurat VlnPlot function to create violin plots that visualize the distribution of gene expression levels across different cell clusters. We verified cell-type annotation with 2 methods. Our annotations were checked with the labels provided by original publication and with the automated cell-type labeling algorithm in SingleR. SingleR is an automatic, referenced-based annotation method for scRNA-seq data; it labels cells from the CHP dataset based on similarity to the referenced dataset. SingleR contains built-in references for automatic cell type labeling. We used the mouse reference dataset to label the ChP data as a form of verification. We also analyzed gene expression in normal human choroid plexus with a snRNA-seq database (38).

The mouse SFO single-cell transcriptomic dataset was from GEO: GSE154048 (56). We removed cells with a mitochondrial percentage greater than 7% and cells with more than 6,000 genes. Normalization, clustering, and downstream analysis were conducted with the pipeline described earlier.

The mouse brain median eminence transcriptomic dataset was from a published study (GEO: GSE160519) (36). The raw counts data were processed in R Seurat packages (version: 4.3.0) for quality control, normalization, clustering, and downstream analyses. Cells with fewer than 200 detected genes or a mitochondrial gene percentage greater than 10% were removed. For the remaining cells, a scale factor of 10,000 reads/cell was used to normalize expression counts. The top 2000 variable genes were identified with the vst method, and the first 30 PCs were used for shared nearest neighbor clustering and UMAP analyses. The EC subset was subjected to subcluster analyses. The processed Seurat object was visualized in a shiny database with the ShinyCell package (version: 2.1.0). The code that we used to perform the scRNA-seq analysis is available from the corresponding author upon request.

The human brain choroid plexus snRNA-seq dataset (GEO: GSE264154) was from a published study (38). The raw data of 3 disease-free human choroid plexus samples were processed in R Seurat packages (version: 4.3.0). To identify differentially expressed genes between different cell types, the FindMarkers function in Seurat was used, which applies a Wilcoxon Rank Sum test and performs multiple test correction with the Bonferroni method. A corrected *P*-value < 0.05 was set as cutoff for significance.

### RT-PCR studies.

ChP (micro-dissected from the 3V, 4V, and LV and pooled together), cerebral cortex, and heart were isolated from 2–3-month-old WT mice, snap frozen, and then put into TRIzol buffer (Thermo Fisher Scientific) for RNA isolation. Total RNA was extracted with the RNeasy kit (Qiagen) and treated with DNase I (Ambion). RNA was reverse transcribed with random primers and the SuperScript III cDNA Synthesis Kit (Invitrogen). RT-PCR reactions were performed on a QuantStudio5 system (Thermo Fisher Scientific) with SYBR Green PCR Master Mix (Bioland). Transcript levels were calculated by the comparative cycle threshold method. Oligonucleotide primers are listed in Supplemental Table 2.

### Assessing TRL margination along capillaries by electron microscopy.

WT mice were injected with 5 μl of 51 mM tetrahydrolipstatin (THL). After 2 min, mice were given an IV injection of TRLs (500 μg TRL protein in a volume of 250 μl) and 5 μl of 51 mM THL via the vena cava. After 5 min, mice were perfused through the left ventricle with 20 ml of perfusion buffer (pH 7.4) containing 0.25% LaCl_3_ and DyCl_3_ (Millipore Sigma, 203521-25G and 289272-25G), 0.1 M sodium cacodylate, 50 mM sucrose, 2 mM CaCl_2_, and 1 mM MgCl_2_ followed by perfusion-fixation with 20 ml of perfusion buffer containing 2% (vol/vol) PFA and 2% (vol/vol) glutaraldehyde (Electron Microscopy Sciences, 16222). The brain was excised and immersed in the same fixative solution. After overnight fixation, the brain was sliced into 100 μm-thick sections with a vibratome; slices containing the ChP were selected, trimmed, and prepared for electron microscopy (11). Vibratome-sliced tissue sections were removed from fixative and washed in 3 5-min washes of 0.1 M sodium cacodylate buffer (pH 7.4). Tissues were then incubated in 0.1 M sodium cacodylate buffer containing 0.2 M imidazole for 1 h. The imidazole solution was removed, and tissues were subjected to 2 rounds of osmication. First, tissues were incubated in 0.1 M sodium cacodylate buffer containing 2% osmium tetroxide (OsO_4_; Electron Microscopy Sciences, 19150) for 45 min. This solution was then removed and replaced with 0.1 M sodium cacodylate buffer containing 2% osmium tetroxide and 1.5% potassium ferricyanide for an additional 45 min. Following osmication, tissues were rinsed with 3 5-min washes of deionized water. Tissue sections were then incubated in 2% uranyl acetate (Electron Microscopy Sciences, 22400) at 4°C overnight. On the next day, tissues were rinsed in 3 5-min washes of deionized water and then dehydrated through a graded acetone series. This series included 5-min washes in 50%, 70%, 80%, and 90% acetone, followed by 2 5-min washes in 100% acetone. The tissues were then preinfiltrated with a mixture of 67% acetone and 33% resin (prepared from: 20 mL Embed 812, 9 mL DDSA, 12 mL NMA, 10 mL BDMA; Electron Microscopy Sciences, 14121) overnight. This was followed by 2 exchanges in 100% resin totaling 2 h. The tissue slices were placed between 2 aclar films (Electron Microscopy Sciences, 50425-10) and polymerized at 60°C for 48 h. Resin-embedded slices were excised from between the aclar sheets and glued to empty resin blocks using cyanoacrylate adhesive (Electron Microscopy Sciences, 12687-01) with the tissue oriented for coronal sectioning. Ultrathin sections were obtained using a Leica Ultracut ultramicrotome, with a section thickness of ~ 70 nm for TEM and 250 nm for SEM. Sections for TEM were collected onto glow-discharged 300 mesh formvar-coated copper grids (Electron Microscopy Sciences, FF300-Cu-50), while sections for SEM were collected onto glow-discharged silicon wafers (Electron Microscopy Sciences, 71893-10). Images were acquired using the low-voltage MiniTEM (Vironova AB) or a Helios UC5 DualBeam Scanning Electron Microscope (Thermo Fisher). The SEM was operated at an accelerating voltage of 5 kV, a spot size of either 0.4 or 0.8 nA, and a working distance of ~ 4 mm with the concentric backscatter detector. Images were acquired at various magnifications between 8,000 and 35,000.

### NanoSIMS imaging.

To prepare [²H]TRLs, *Gpihbp1^–/–^* mice were fed a fat-free diet (62% sucrose; Envigo, TD.03314) for 13 days and then given uniformly labeled [^2^H]mixed fatty acids (Cambridge Isotope Laboratories, DLM-8572-PK) by gastric gavage every 12 h for 4.5 days. [²H]TRLs were prepared from the plasma by ultracentrifugation (11). WT mice were injected with [^2^H]TRLs (300 μg TRLs in 200–300 μl PBS) through the inferior vena cava. After 5 min, the mice were perfused with 5 ml of HEPES/saline buffer (pH 7.2) followed by perfusion-fixation with 10 ml of a HEPES buffer (50 mM, pH 7.2) containing 2.5% (vol/vol) glutaraldehyde. Tissue sections were embedded in resin, trimmed, and sectioned with a Leica UC7 ultramicrotome. 500-nm-thick sections were mounted on silicon wafers and analyzed with a NanoSIMS 50L instrument (CAMECA). A 16-KeV ^133^Cs^+^ beam was used to bombard the sample, and secondary electrons (SEs) and secondary ions (^1^H^–^, ^2^H^–^,^12^C^–^) were collected. Regions-of-interest were presputtered with an approximately 1.1-nA beam current (primary aperture D1=1) to remove the platinum coating and to implant ^133^Cs^+^ to a dose of approximately 1 × 10^17^ ions/cm^2^ to achieve optimal secondary ion release. 27 27-μm regions were imaged with an approximately 2 pA beam current (primary aperture D1=2) and a dwell time of 5 ms/pixel per frame. Multiple frames and 256 256-pixel images were obtained. The images were adjusted for contrast with the OpenMIMS plugin (MIMS, Harvard University), and distortions across multiple frames were corrected with NanoSIMS Stabilizer (57) in ImageJ.

### Statistics.

For single cell transcriptomics, raw data were processed in R Seurat packages (version: 4.3.0). To identify differentially expressed genes between different cell types, the FindMarkers function in Seurat was used, which applies a Wilcoxon Rank Sum test and performs multiple test correction with the Bonferroni method. A corrected *P* value < 0.05 was set as cutoff for significance. For the data in Supplemental Figures 2, 3, and 17, data were presented as mean ± SEM and analyzed by ANOVA or Student’s *t* test. All statistical tests are outlined in Methods and the figure legends.

### Data availability.

We reanalyzed scRNA-seq databases and snRNA-seq databases published by others and have cited the publications and the public repositories. All other data are included in the manuscript and supplemental files.

## Author contributions

WS, LPN, JC, SGY, and LGF designed the experiments and wrote the paper. WS, MH, LPN, TLL, MH, APT, EK, JC, PK, HJ, YT, SDM, CKW, AMP, JS, JPK, JK, and YY performed, collected, and assembled the experiments. WS, JC, SGY, LGF, CB, LH, and MP analyzed data. SGY, LGF, APB, MP, WS, CB, MAM, and HJ secured funding.

## Supplementary Material

Supplemental data

Supporting data values

## Version 1 10/01/2025

Electronic publication
