# Supplementary material for: GPIHBP1, lipoprotein lipase, and triglyceride-rich lipoproteins in capillaries of the choroid plexus and circumventricular organs
Source: J Clin Invest. 2025 Oct 1;135(19):e191867. doi: 10.1172/JCI191867 (PMC12483605; doi:10.1172/JCI191867)
Supplement: Supplemental data [file jci-135-191867-s212.pdf]

Supplemental Figures for:

GPIHBP1, lipoprotein lipase, and triglyceride-rich lipoproteins in capillaries of the choroid plexus and circumventricular organs

Wenxin Song[1], Madison Hung[1], Ellen Kozlov[1], Megan Hung[1], Anh P. Tran[1], James Carroll[1], Le Phoung Nguyen[1], Troy L. Lowe[1], Paul Kim[1], Hyesoo Jung[1], Yiping Tu[1], Joonyoung Kim[1], Ashley M. Presnell[1], Julia Scheithauer[1], Jenna P. Koerner[1], Ye Yang[1,2], Shino D. Magaki[3], Christopher K. Williams[3], Michael Ploug[4,5], Haibo Jiang[6], Christer Betsholtz[7,8], Maarja Andaloussi Mäe[7], Liqun He[7], Anne P. Beigneux[1], Loren G. Fong[1,†], and Stephen G. Young[1,2,†]

[1]Department of Medicine and [2]Department of Human Genetics, David Geffen School of Medicine, UCLA, Los Angeles, CA, USA, [3]Department of Pathology and Laboratory Medicine, David Geffen School of Medicine, UCLA, Los Angeles, CA, USA; [4]Finsen Laboratory, Copenhagen University Hospital - Rigshospitalet, DK–2200 Copenhagen N, Denmark; [5]Biotechnology Research and Innovation Centre (BRIC), University of Copenhagen, DK-2200 Copenhagen N, Denmark; [6]Department of Chemistry, The University of Hong Kong, Hong Kong, China; [7]Department of Immunology, Genetics, and Pathology, Rudbeck Laboratory, Uppsala University, Uppsala, Sweden; [8]Department of Medicine-Huddinge, Karolinska Institute Campus Flemingsberg, Huddinge, Sweden.

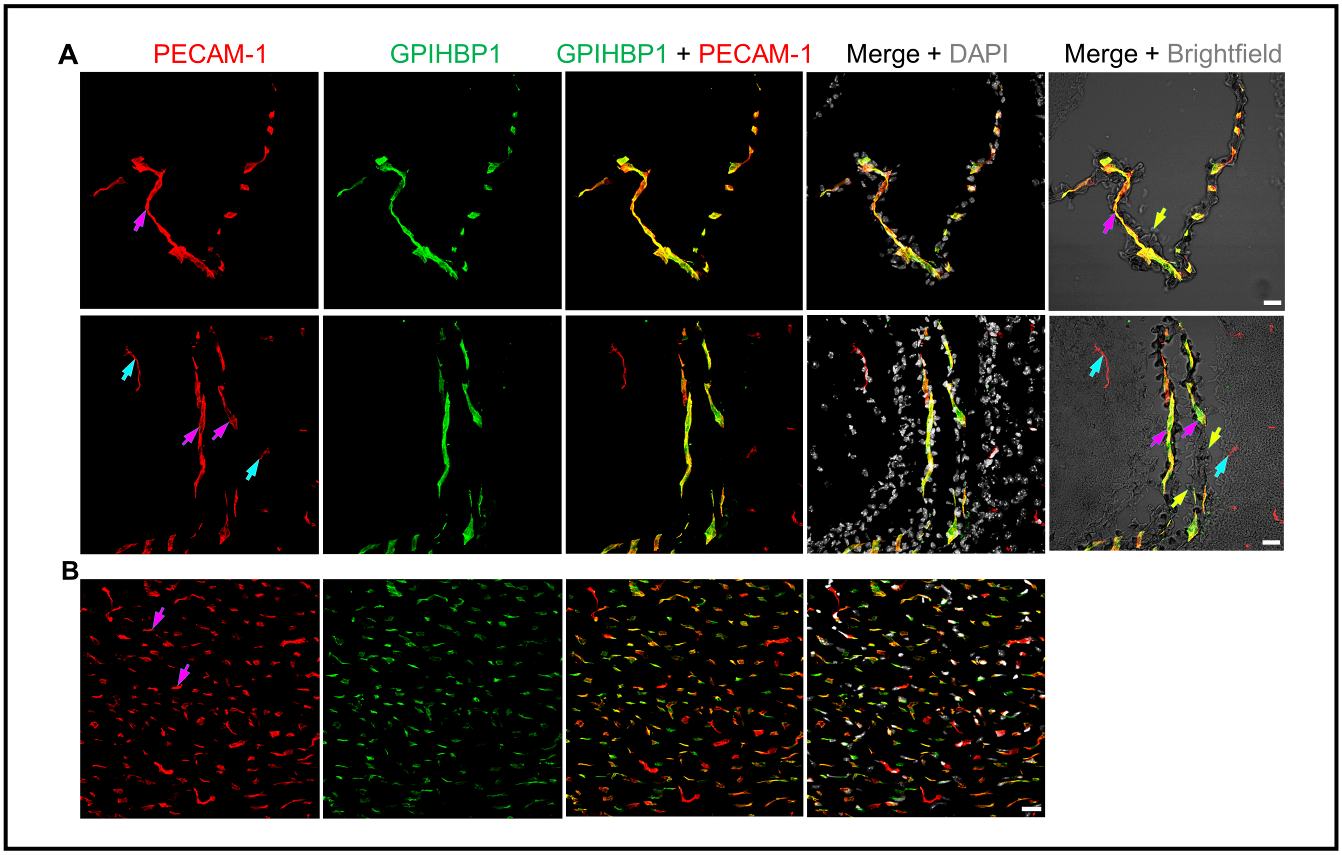

Supplemental Figure 1. Confocal micrographs revealing GPIHBP1 in PECAM-1–positive capillary endothelial cells (ECs). Sections of the choroid plexus (ChP) (**A**) and heart (**B**) were incubated with mAb 11A12 (against GPIHBP1) and rabbit polyclonal antibody against PECAM-1. Ab binding was detected with Alexa Fluor–labeled secondary antibodies. In ChP and heart, mAb 11A12 bound to the PECAM-1–positive capillaries (*purple* arrows) but did not bind to the PECAM-1–positive capillaries in the brain parenchyma (*blue* arrows). Brightfield images made it possible to visualize ChP epithelial cells (*yellow* arrows). Scale bar, 20 µm.

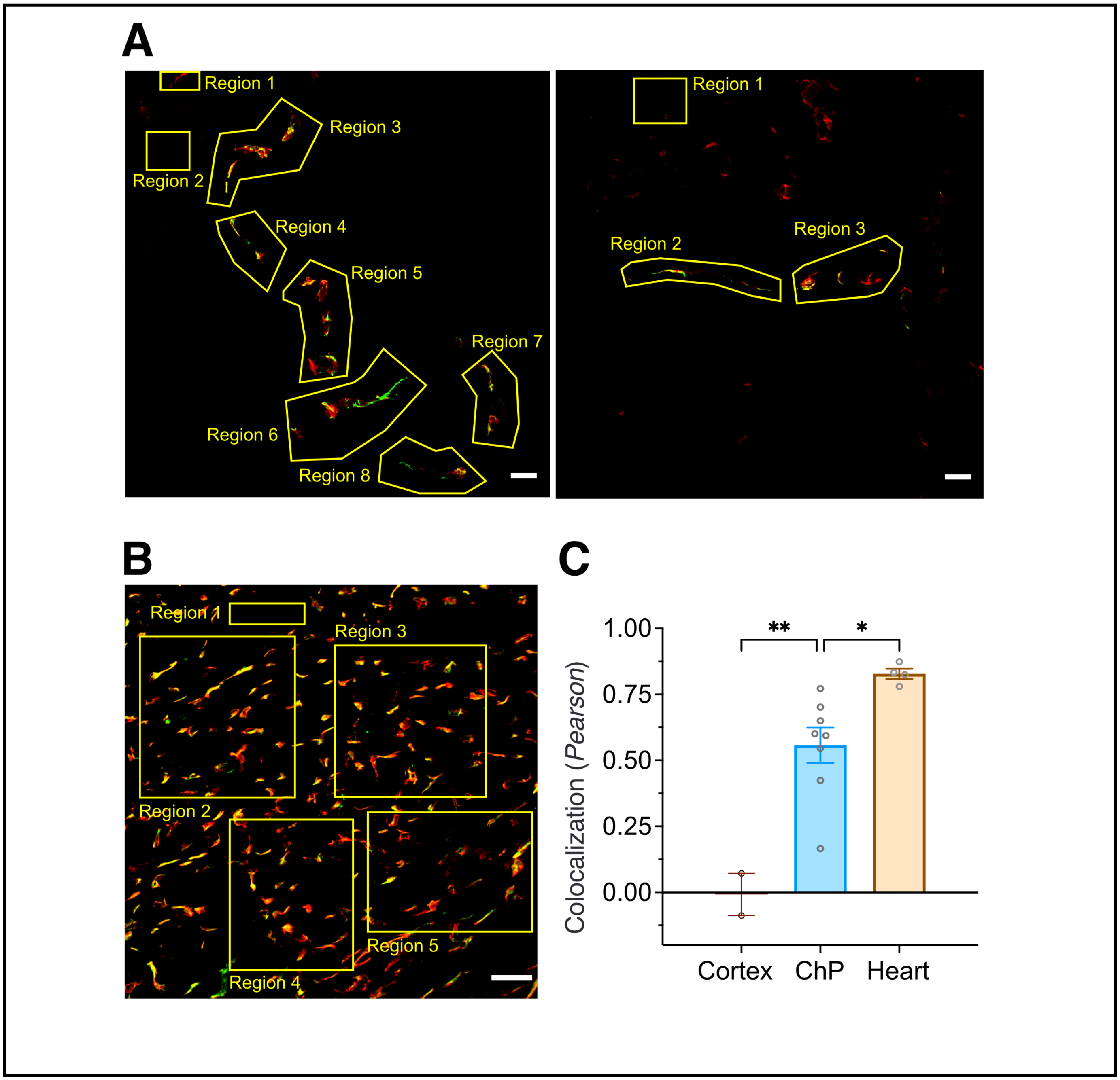

Supplemental Figure 2. Pearson correlation studies to assess colocalization of GPIHBP1 (green) and PECAM-1 (red) signals in the merged ChP panel and the merged heart panel in Figures 1A and 1B, respectively. (**A**) The image on the left is taken from the top image in Figure 1A. Eight regions are circled; regions 1 and 2 are from cerebral cortex, and regions 3–8 are from ChP. The image on the right is from the bottom image in Figure 1A. Three regions are circled; region 1 is from cerebral cortex, and regions 2–3 are from ChP. (**B**) The image of the heart is taken from Figure 1B. Five regions are circled; region 1 lacks capillaries, and regions 2–5 contain many capillary segments. (**C**) Pearson correlation coefficients, reflecting colocalization of GPIHBP1 and PECAM-1 signals, in cerebral cortex, ChP, and heart. Data are presented as mean ± SEM and were analyzed by one-way ANOVA. $^{**}p < 0.01$; $^{*}p < 0.05$.

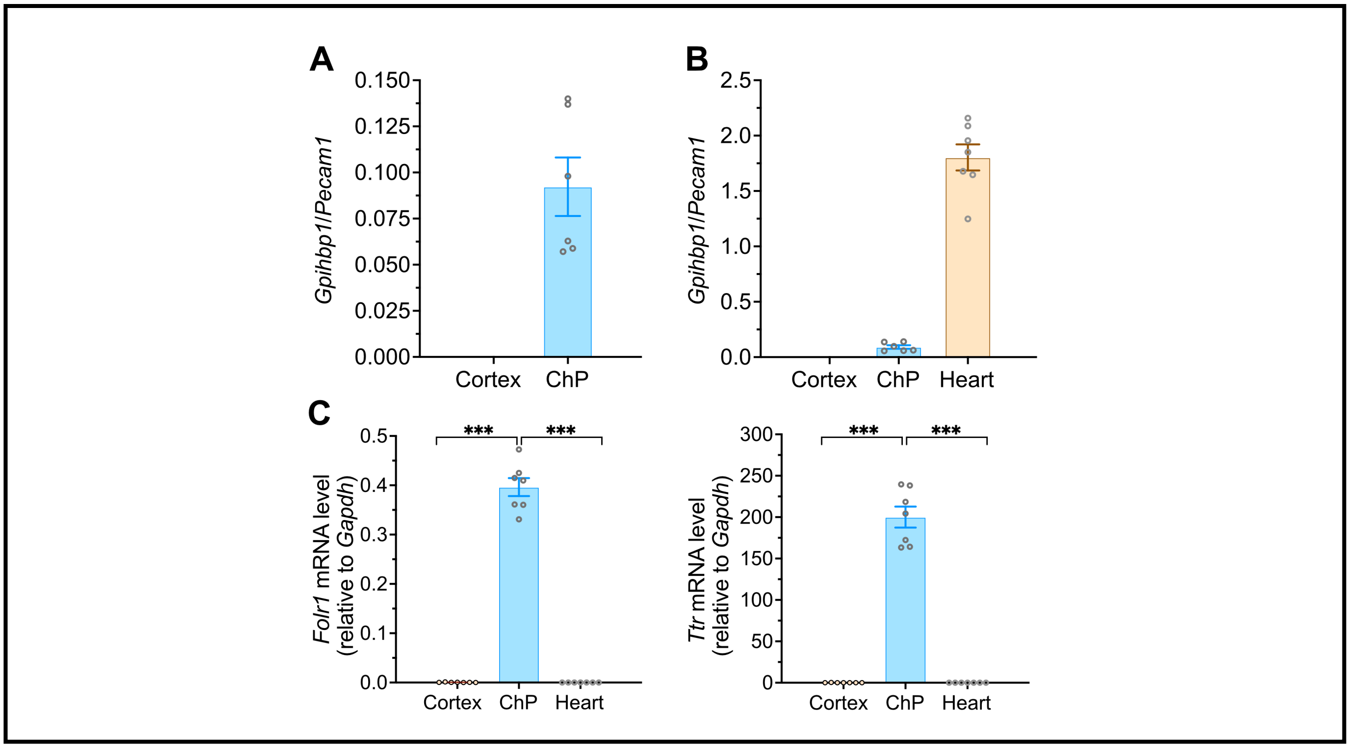

Supplemental Figure 3. *Gpihbp1* transcripts were detected by RT-PCR in the mouse ChP and heart. RNA was prepared from ChP micro-dissected from WT mice (n = 6–7); RNA was also prepared from the apex of the heart and the cerebral cortex. (**A**) *Gpihbp1* transcripts were detected in the ChP but were absent from the cerebral cortex. (**B**) *Gpihbp1* transcripts, relative to *Pecam1* transcripts, were lower in the ChP than in the heart. (**C**) *Fol1r* and *Ttr* transcripts (markers of ChP epithelial cells) were detected in the ChP but not heart or cerebral cortex. Data are presented as mean ± SEM and were analyzed by one-way ANOVA. ***P < 0.001.

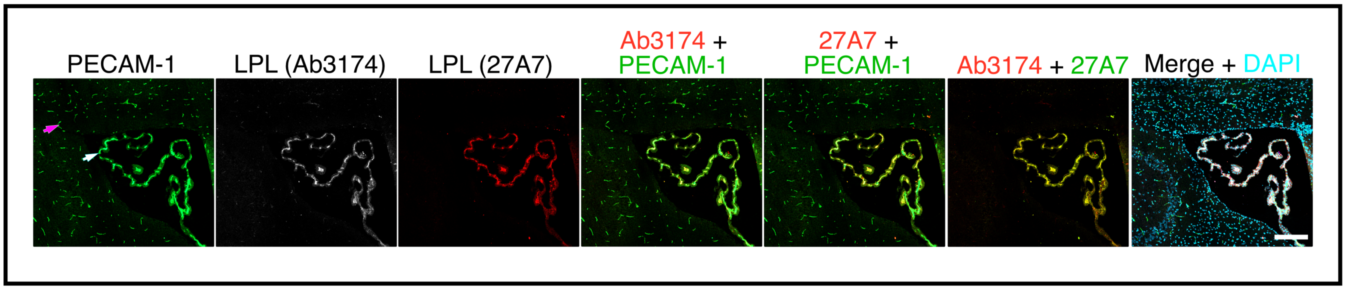

Supplemental Figure 4. LPL is located on the luminal surface of capillaries of the ChP but not capillaries of the surrounding brain parenchyma. WT mice were given an IV injection of Alexa Fluor 647–Ab3174 (*white*), Alexa Fluor 555–27A7 (*red*), and Alexa Fluor 488–2H8 (against PECAM-1, *green*). After 2 min, the vasculature was perfused extensively with PBS, and tissue sections were prepared for microscopy. The LPL-specific antibodies bound to capillaries of the ChP but not capillaries of the surrounding brain parenchyma; mAb 2H8 bound to the capillaries of both ChP (*white* arrow) and brain parenchyma (*purple* arrow). Scale bar, 200 µm.

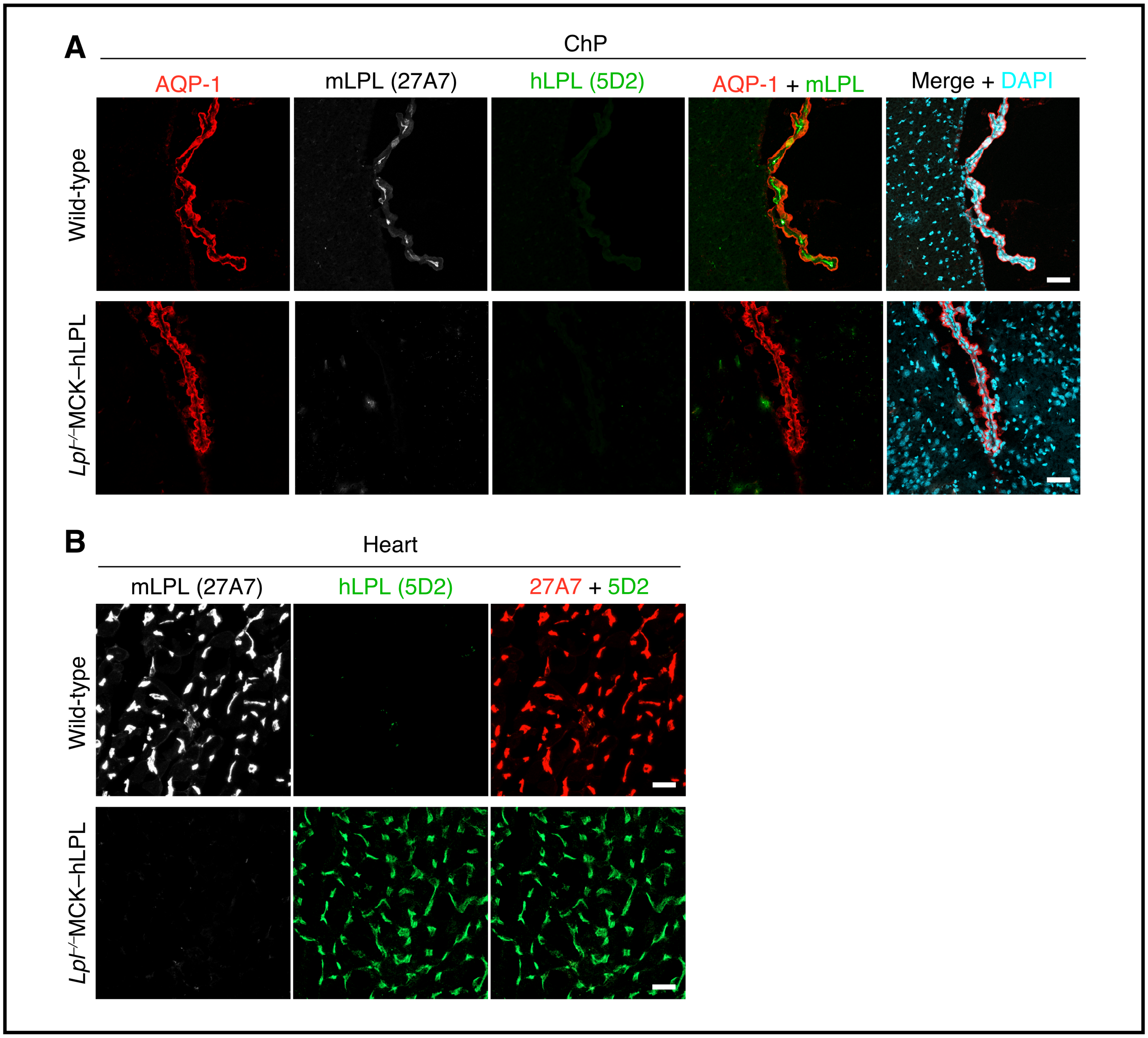

Supplemental Figure 5. LPL is located along the luminal surface of ChP capillaries of WT mice but not *Lpl*$^{-/-}$MCK–hLPL mice. WT mice and *Lpl*$^{-/-}$MCK-hLPL mice were given an IV injection of Alexa Fluor 555–27A7 [against mouse LPL (mLPL), *white*] and Alexa Fluor 488–5D2 [against human LPL (hLPL), *green*]. After 2 min, the vasculature was perfused with PBS, and tissue sections were prepared for microscopy. Confocal micrographs of the ChP (**A**) and heart (**B**) were prepared to assess intracapillary binding of mAb 5D2 [which detects hLPL but not mLPL)] and mAb 27A7 (which detects mLPL but not hLPL) to capillaries. To detect ChP epithelial cells, sections were stained with an AQP-1 mAb (*red*). mAb 5D2 did not detect hLPL in the ChP capillaries of WT or *Lpl*$^{-/-}$MCK-hLPL mice; mAb 5D2 detected hLPL in the heart of *Lpl*$^{-/-}$MCK-hLPL but not in the heart of WT mice. mAb 27A7 detected mLPL in ChP and heart capillaries of WT mice but not *Lpl*$^{-/-}$MCK-hLPL mice. Images were recorded with a 20× objective. Scale bar, 50 μm.

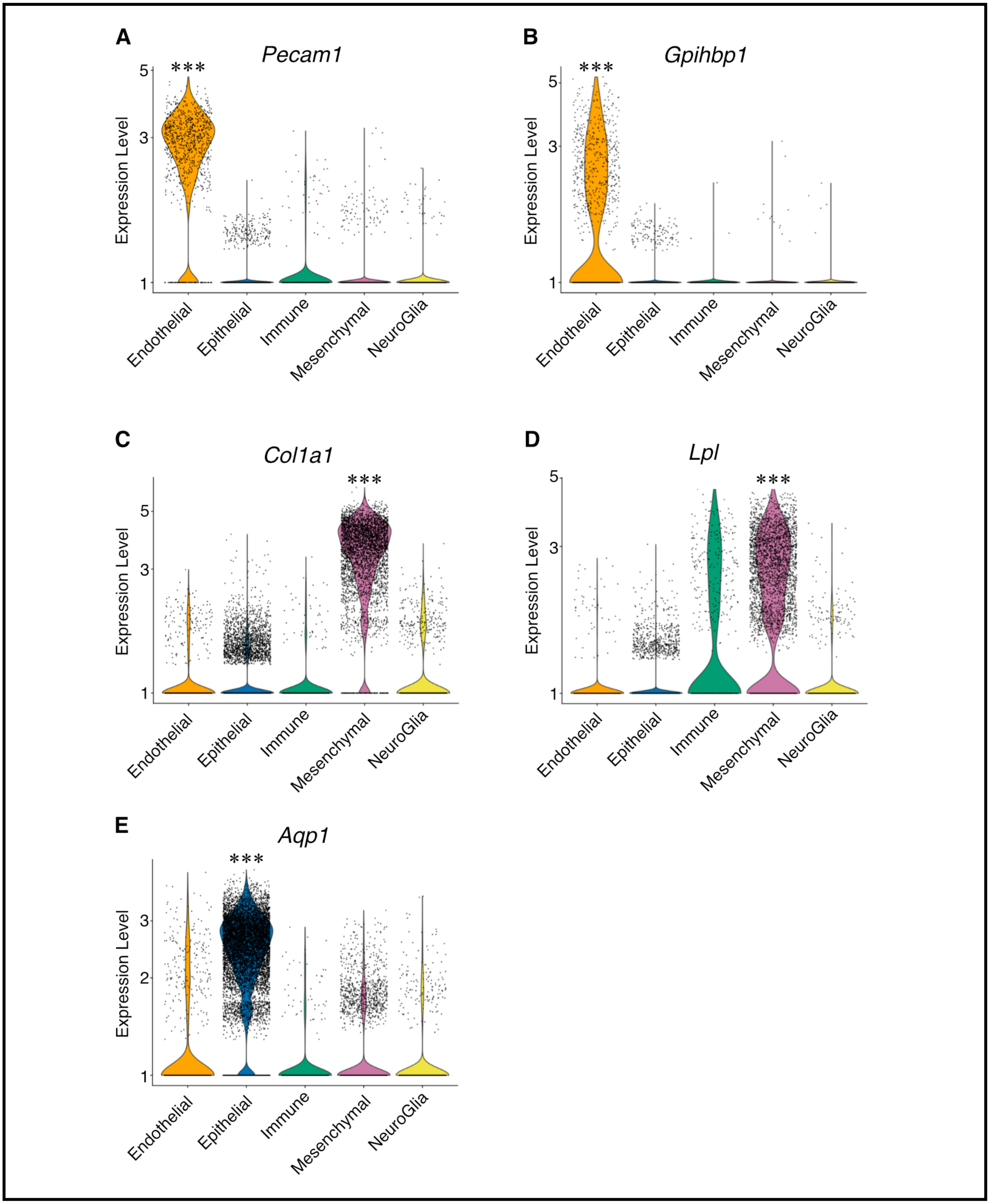

Supplemental Figure 6. Violin plots depicting *Gpihbp1, Pecam1*, *Lpl*, *Col1a1*, and *Aqp1* expression in the ChP based on a scRNA-seq dataset from E16.5 mouse embryos (1). *P* values and $\log_2$ fold-

change (avg_ $\log_2$FC) for marker genes (*Pecam1*, *Gpihbp1*, *Col1a1*, *Lpl*, *Aqp1*) were identified by differential expression analysis with the Seurat FindMarkers function with default parameters and the nonparametric Wilcoxon rank-sum test. The adjusted p values were adjusted with the Bonferroni correction method. (**A**) *Pecam 1* was >7.9 $\log_2$ fold-change higher in endothelial cells than in the combination of all other cell clusters (Bonferroni-adjusted $p \leq 2.23 \times {}^{-308}$), confirming endothelial cell specificity. (**B**) *Gpihbp1* is >8.9 $\log_2$ fold-change higher in endothelial cells than in the combination of all other cell clusters (Bonferroni-adjusted $p \leq 2.23 \times 10^{-308}$), confirming endothelial cell specificity. (**C**) *Lpl* is >4.5 $\log_2$ fold-change higher in mesenchymal cells than in the combination of all other cell clusters (Bonferroni-adjusted $p \leq 2.23 \times 10^{-308}$), confirming mesenchymal cell specificity. (**D**) *Col1a1* is >6.2 $\log_2$ fold-change higher in mesenchymal cells than in the combination of all other cell clusters (Bonferroni-adjusted $p \leq 2.23 \times 10^{-308}$), confirming mesenchymal cell specificity. (**E**) *Aqp1* is >3.5 $\log_2$ fold-change higher in epithelial cells than in the combination of all other cell clusters (Bonferroni-adjusted $p \leq 2.23 \times 10^{-308}$), confirming epithelial cell specificity.

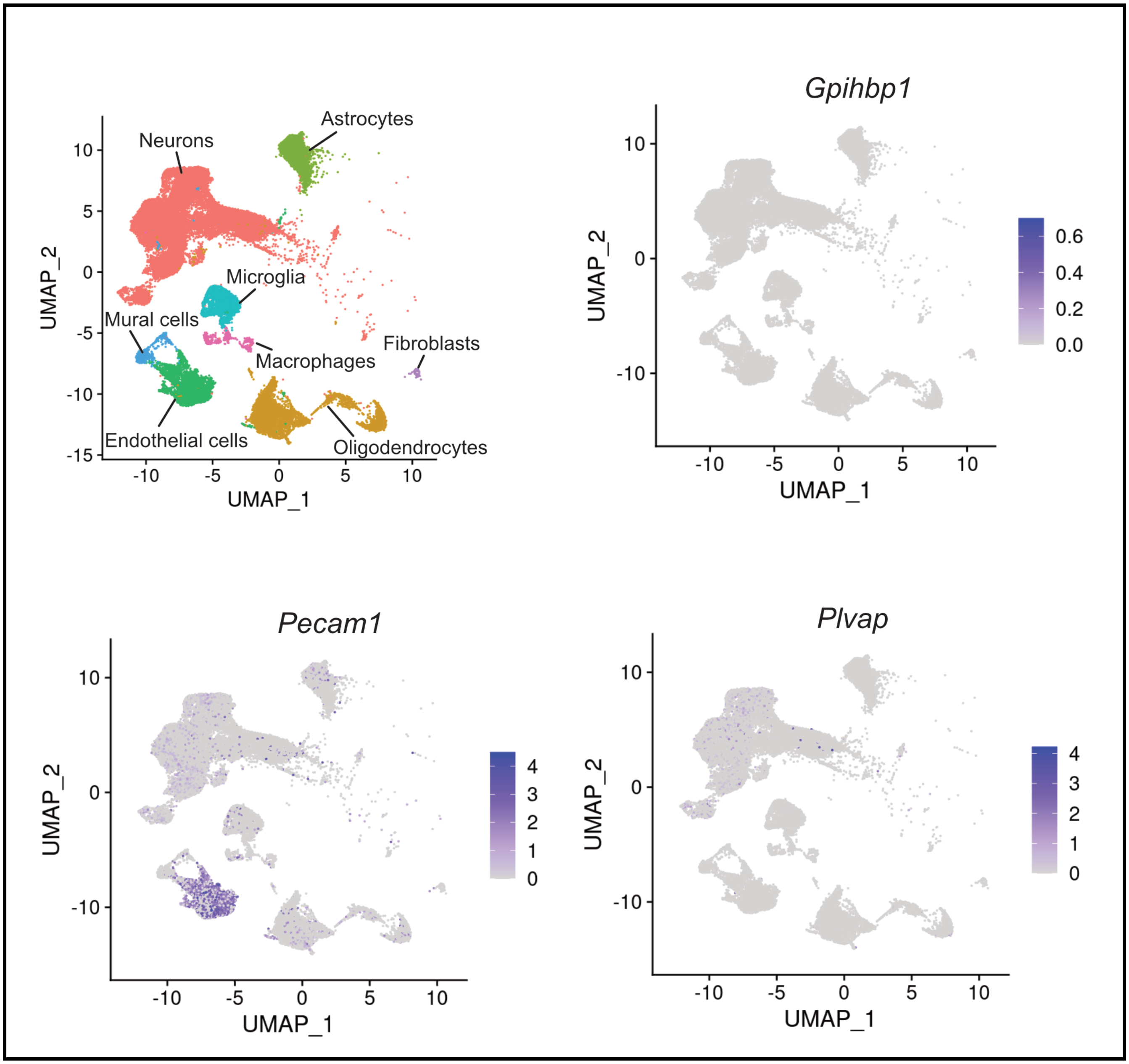

Supplemental Figure 7. *Gpihbp1* is not expressed in ECs of the mouse cerebral cortex. UMAP of cell profiles from scRNA-seq studies of the somatosensory cortex in 9-week-old WT mice (2). *Pecam1* transcripts were in ECs of the cerebral cortex, whereas *Gpihbp1* and *Plvap* transcripts were virtually absent.

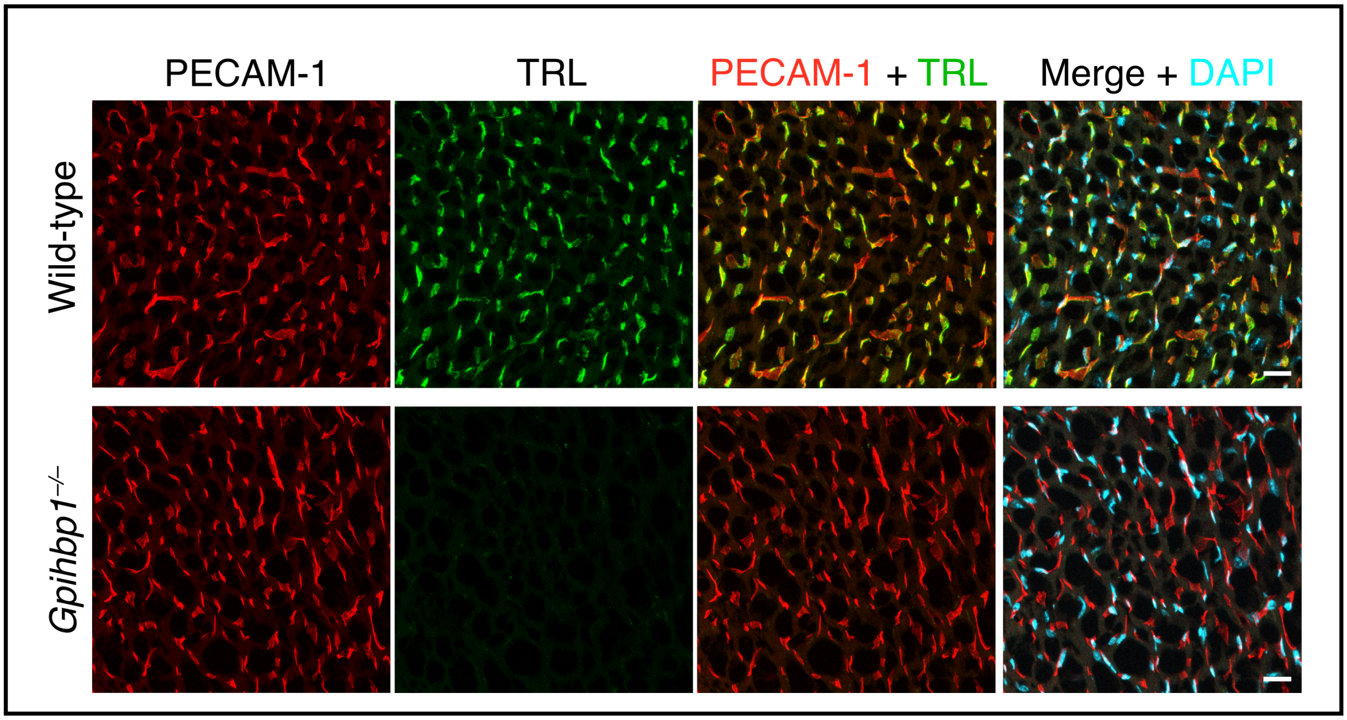

Supplemental Figure 8. Confocal micrographs documenting margination of triglyceride-rich lipoproteins (TRLs) along the luminal surface in heart capillaries of WT mice but not *Gpihbp1*$^{-/-}$ mice. WT and *Gpihbp1*$^{-/-}$ mice were given an IV injection of Alexa Fluor 488–TRLs and Alexa Fluor 555–mAb 2H8 (against PECAM-1), each labeled with an Alexa Fluor dye. After 2 min, the mice were euthanized; the vasculature was perfused extensively with PBS, and sections of the heart were prepared for confocal microscopy. Images were recorded with a 20× objective. Scale bar, 20 µm.

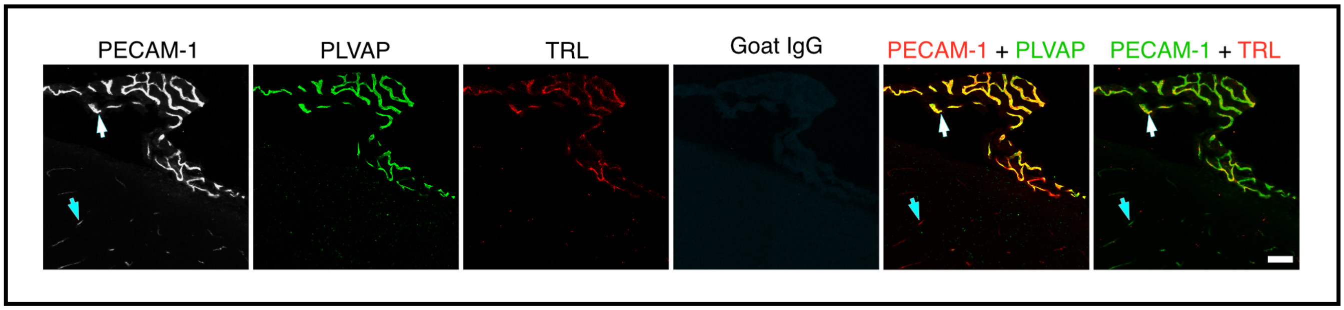

Supplemental Figure 9. TRL margination along capillaries of the ChP but not capillaries of the brain parenchyma. Confocal micrographs of ChP and surrounding brain parenchyma from a WT mouse after an intracarotid artery injection of Alexa Fluor 647–2H8 (against PECAM-1, *white*), Alexa Fluor 555–TRLs (*red*), and Dylight405–goat IgG (*blue*). After 2 min, the vasculature was perfused extensively with PBS and PFA. The absence of goat IgG in the vasculature indicated that the vascular perfusion had been effective. Sections were stained with a PLVAP-specific antibody (a marker of fenestrated ECs, *green*). TRL margination was robust along capillaries of the ChP (*white arrow*) but not in capillaries of the surrounding brain parenchyma (*purple arrow*). Images were recorded with a 20× objective. Scale bar, 50 µm.

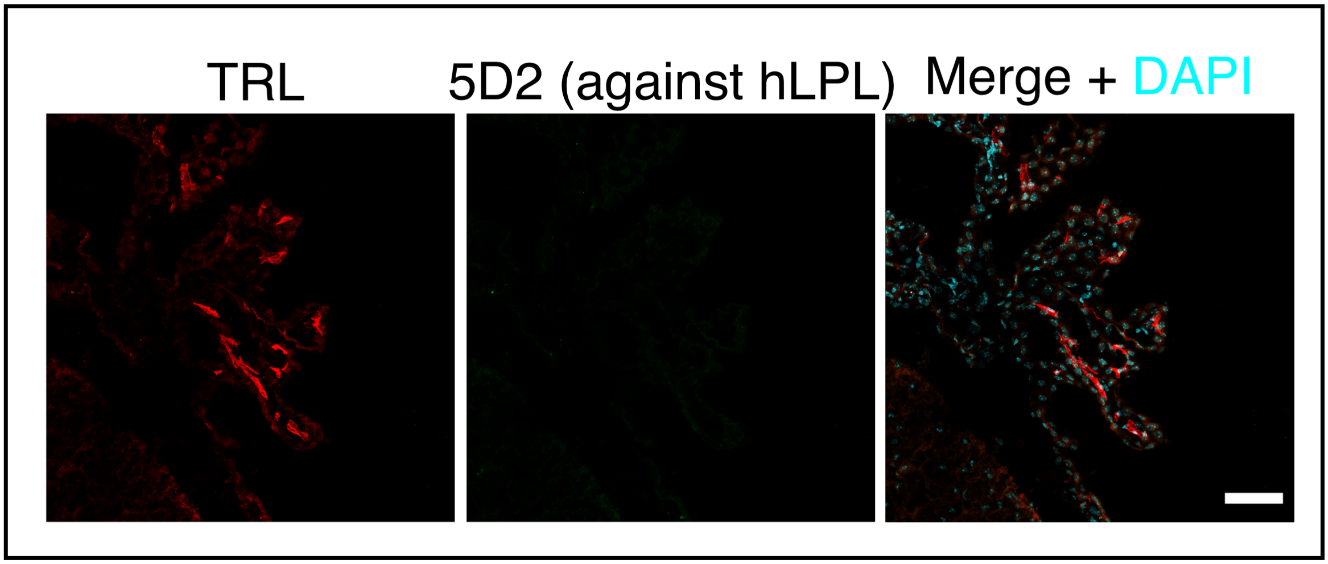

Supplemental Figure 10. Margination of TRLs along ChP capillaries in a WT mouse. Confocal micrographs of ChP from a WT mouse after an intracarotid artery injection of Alexa Fluor 647–TRLs (*red*) and Alexa Fluor 488–5D2 (*green*). mAb 5D2 binds to human LPL but not mouse LPL. After 2 min, mice were perfused extensively with PBS and perfusion-fixed with PFA. The absence of mAb 5D2 in ChP capillaries indicates that the perfusion of the vasculature had effectively removed unbound antibodies. Images were recorded with a 20× objective. Scale bar, 40 μm.

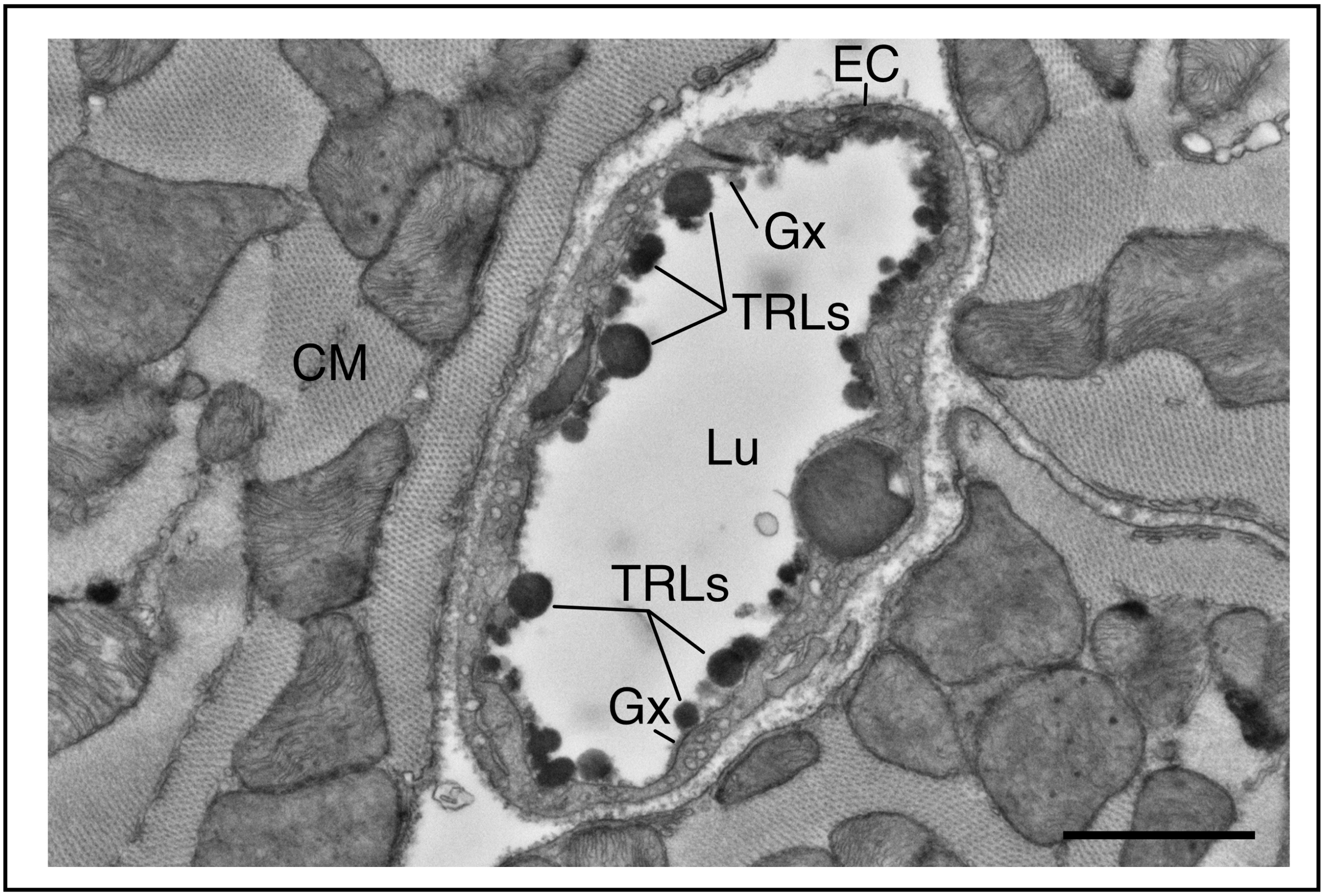

Supplemental Figure 11. Scanning electron micrograph showing margination of TRLs along the luminal surface of a heart capillary after giving WT mice an IV injection of TRLs. The EC glycocalyx was stained with $LaCl_3/DyCl_3$. Lu, lumen; Gx, glycocalyx; EC, endothelial cell; CM, cardiomyocyte. Scale bar, 1 µm.

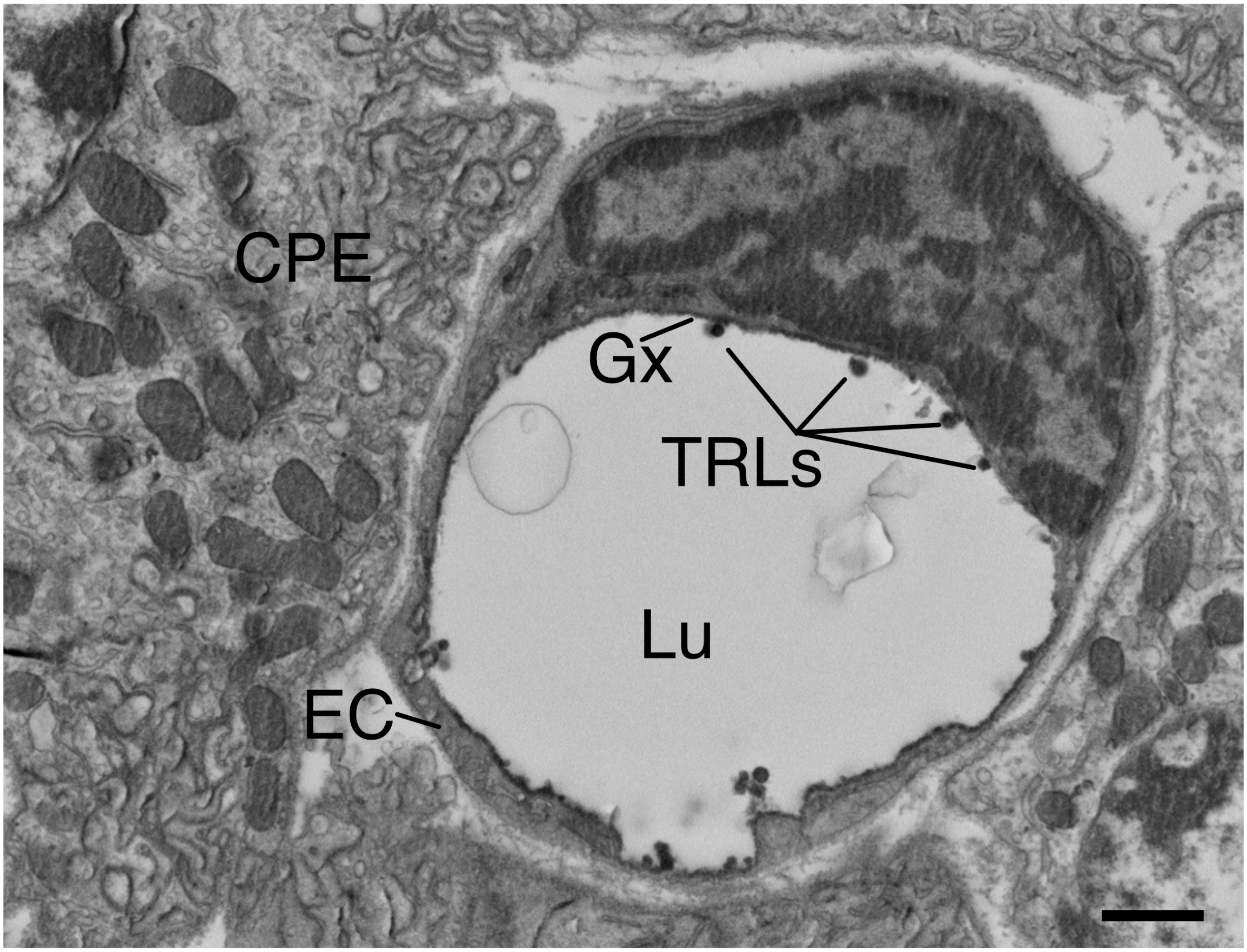

Supplemental Figure 12. Scanning electron micrograph showing margination of TRLs along the luminal surface of a ChP capillary after giving WT mice an IV injection of TRLs. The EC glycocalyx was stained with $LaCl_3$/$DyCl_3$. Lu, lumen; Gx, glycocalyx; EC, endothelial cell; CPE, ChP epithelial cells. Scale bar, 1 μm.

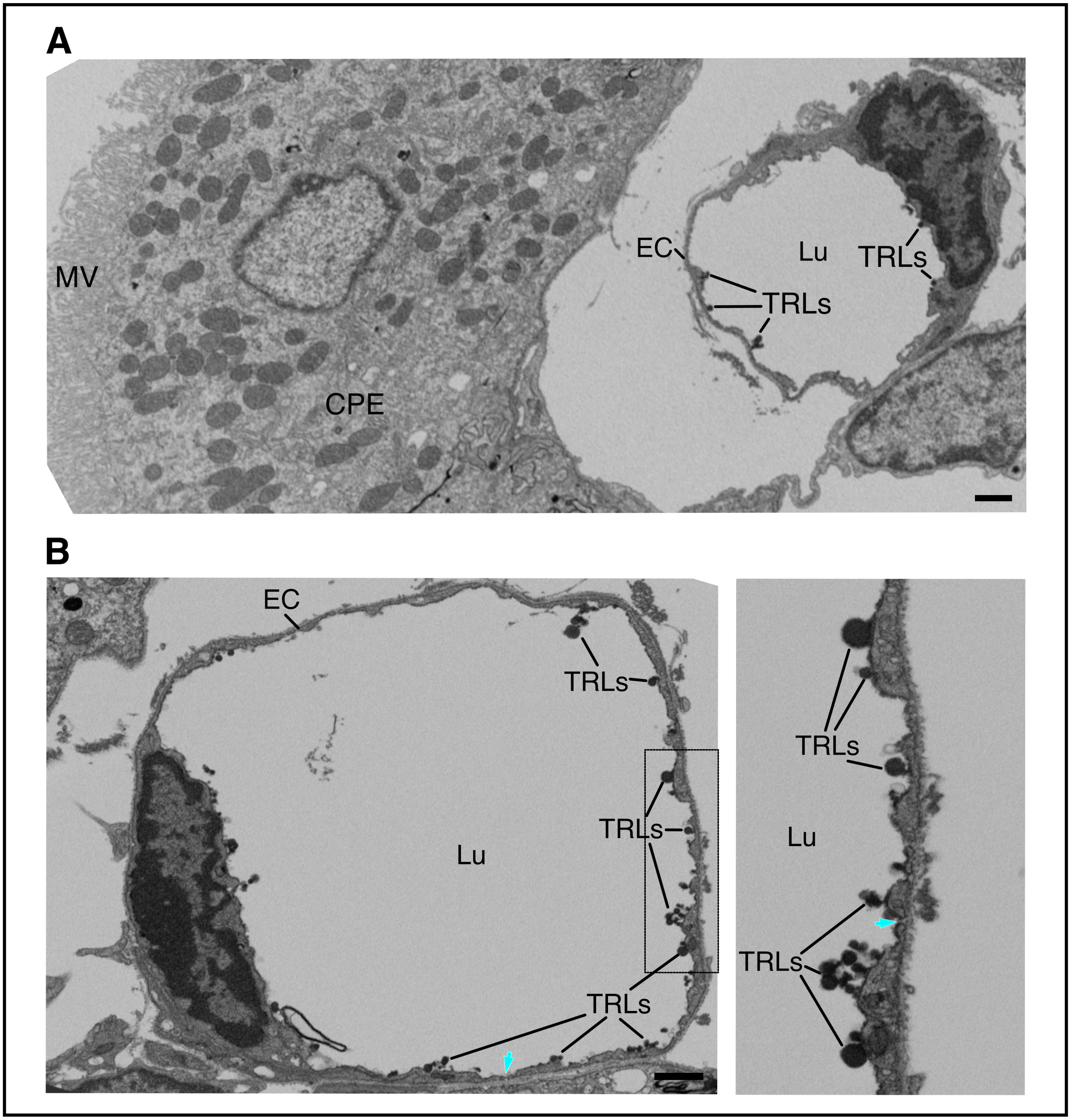

Supplemental Figure 13. Scanning electron micrograph showing margination of TRLs along the luminal surface of a ChP capillary after giving WT mice an IV injection of TRLs. The EC glycocalyx was stained with $LaCl_3/DyCl_3$. (**A**) ChP capillary containing several TRLs as well as an adjacent ChP epithelial cell with abundant microvilli. (**B**) ChP capillary containing several TRLs. The boxed region is shown at a higher magnification on the right. *Blue* arrows point to endothelial cell fenestrations. Lu, lumen; EC, endothelial cell; MV, microvilli; CPE, ChP epithelial cells. Scale bar, 1 µm.

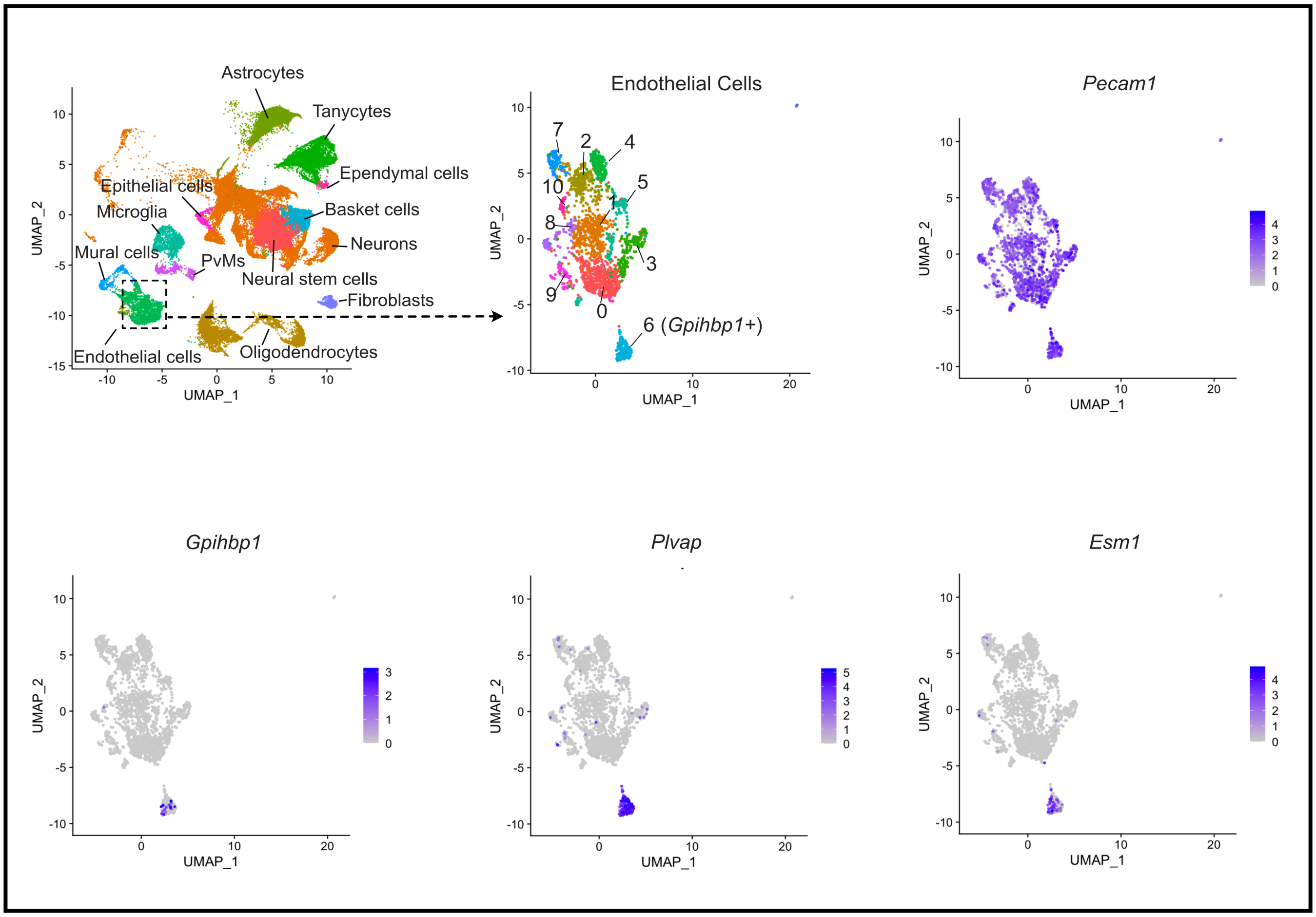

Supplemental Figure 14. *Gpihbp1* is expressed by endothelial cells of fenestrated capillaries in the mouse median eminence (ME). UMAP of cell profiles from scRNA-seq studies of the ME in 9-week-old mice (2) along with a UMAP depicting EC sub-cluster analyses. *Pecam1* transcripts were in all ECs; EC sub-cluster 6 contained transcripts for *Gpihbp1* and markers of fenestrated ECs (*Plvap* and *Esm1*).

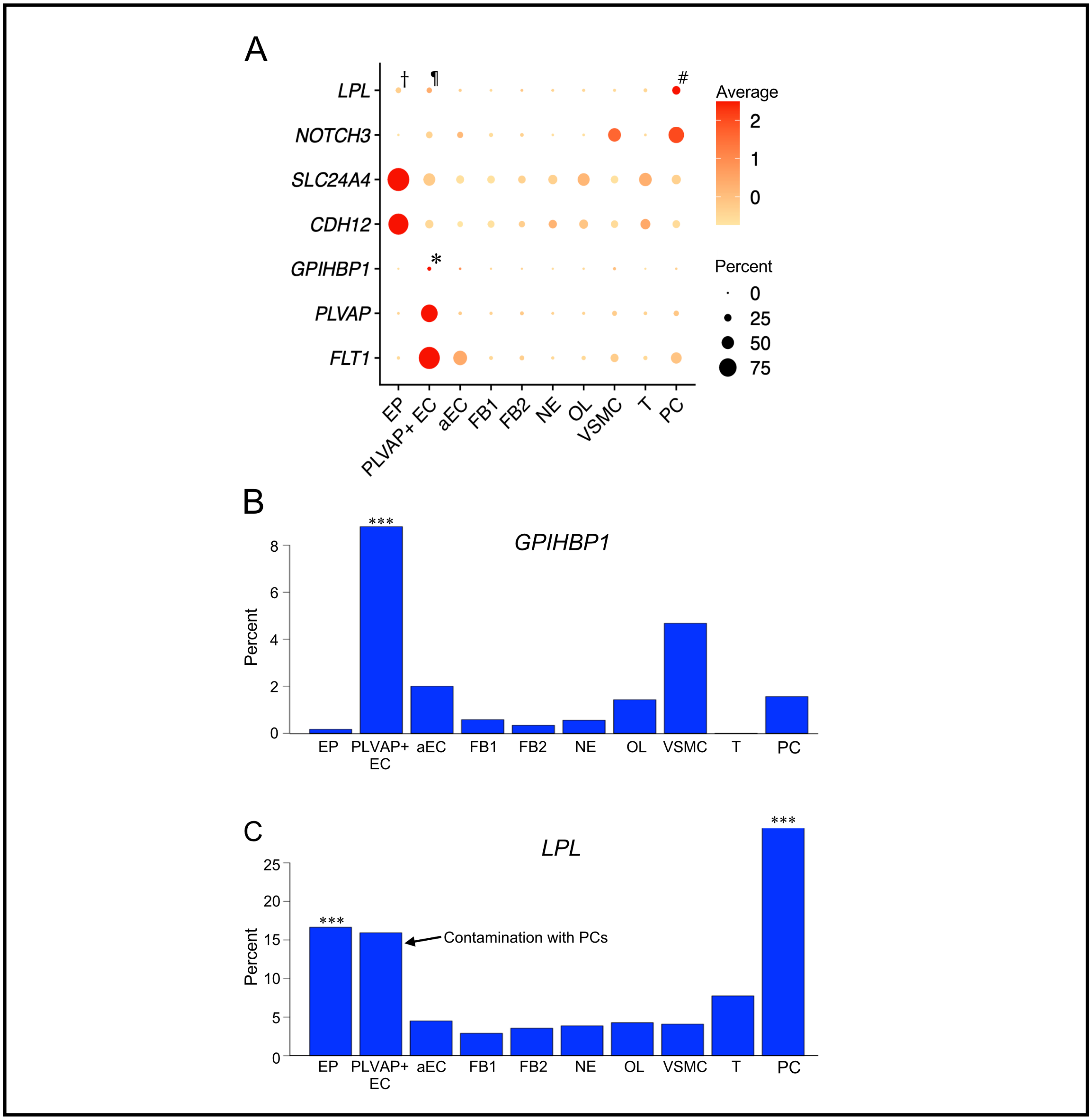

Supplemental Figure 15. *GPIHBP1* and *LPL* expression in disease-free human choroid plexus. (A) Dot-plot depicting average gene expression and percent of cells expressing each gene in choroid plexus epithelial cells (EP), *PLVAP*+ endothelial cells (ECs), arterial ECs (aEC), fibroblasts (FB1, FB2), neurons (NE), oligodendrocytes (OL), vascular smooth muscle cells (VSMC), T cells (T), and pericytes (PC) (3). *GPIHBP1* is expressed prominently in *PLVAP*+ ECs (*); LPL is expressed prominently in PCs (#) and EPs (†). In *PLVAP*+ ECs, LPL expression was almost certainly due to PC contamination (see Supplemental Figure 16). (**B–C**) Bar plots showing the percentage of cells that express *GPIHBP1* and *LPL* in different cell types. *** indicates significant enrichment of *GPIHBP1* and *LPL* expression relative to other cells ($p < 10^{-10}$).

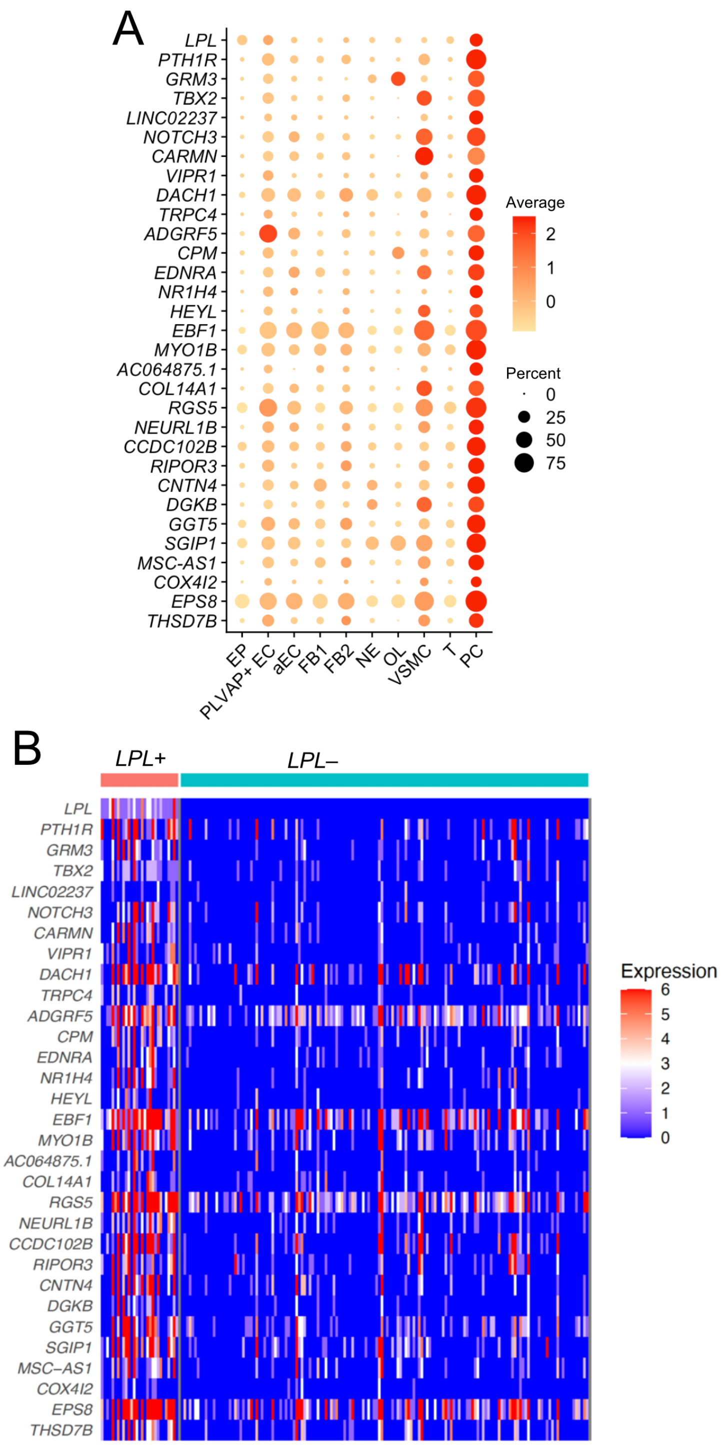

Supplemental Figure 16. The expression of *LPL* in human choroid plexus. (**A**) Dot plot depicting *LPL* expression and the expression of the top 30 pericyte (PC)-enriched genes in epithelial cells (EP), *PLVAP*+ endothelial cells (ECs), arterial ECs (aEC), fibroblasts (FB1, FB2), neurons (NE), oligodendrocytes (OL), vascular smooth muscle cells (VSMC), T cells (T), and pericytes (PC). (**B**) Heatmap showing expression of the top 30 PC genes in *PLVAP*+ ECs with *LPL* transcripts (*LPL*+). The expression of PC-enriched

genes was high in the 29 *LPL*+ *PLVAP*+ ECs and low in the 153 *LPL*– *PLVAP*+ ECs, indicating that the *LPL* reads in *PLVAP*+ ECs were likely due to PC contamination.

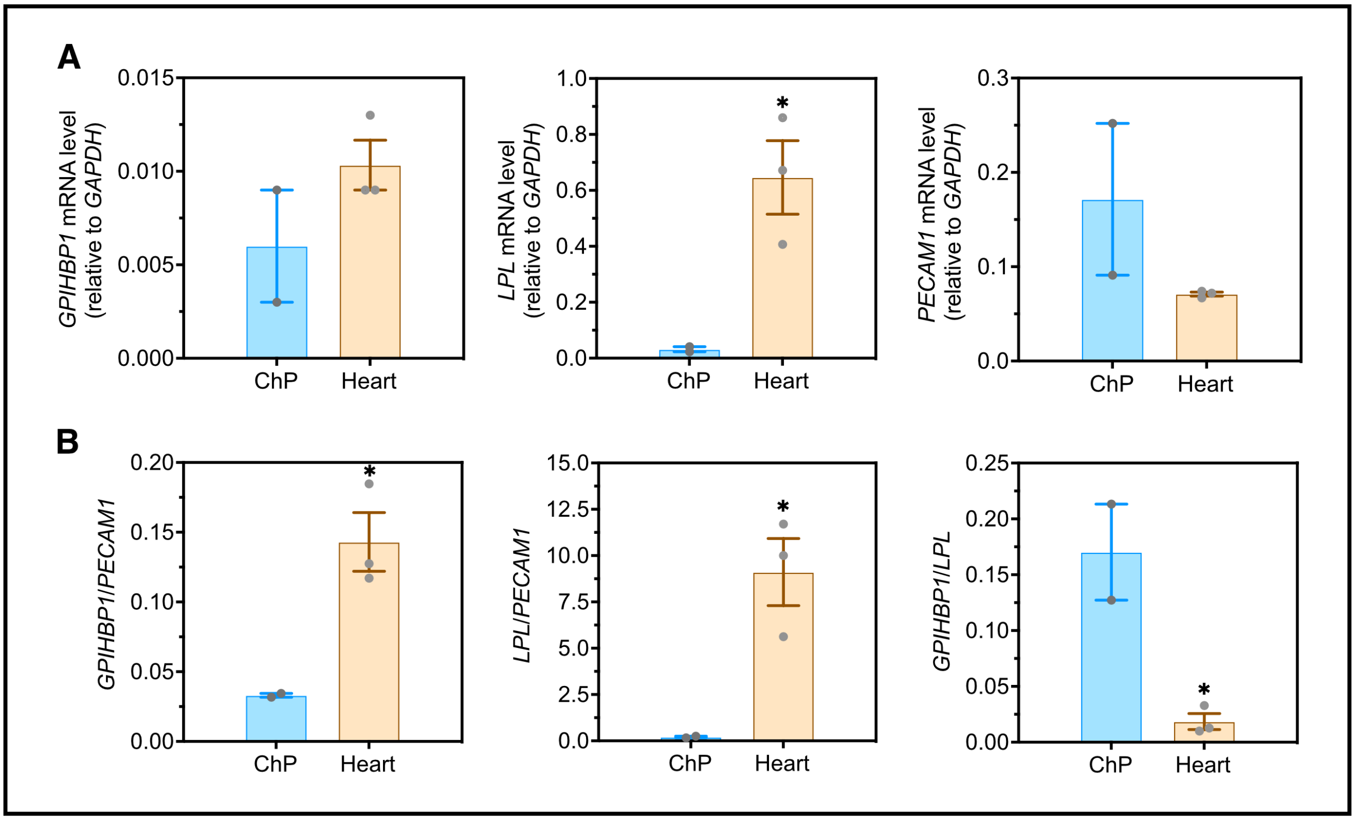

Supplemental Figure 17. *GPIHBP1, LPL,* and *PECAM1* transcripts were assessed by RT-PCR in human ChP and heart. RNA was prepared from archived heart samples (n = 3) and freshly isolated choroid plexus (n = 2) (provided by UCLA's Translational Pathology Core Laboratory). (**A**) Transcript levels for *GPIHBP1*, *LPL*, and *PECAM1* (relative to a commonly used housekeeping gene, *GAPDH*). (**B**) *GPIHBP1/PECAM1, LPL/PECAM1,* and *GPIHBP1/LPL* expression ratios in heart and choroid plexus. Data are presented as mean ± SEM and were analyzed by 2-sided Student's t test. *P < 0.05.

Table S1. Listing of endothelial cell marker gene expression in the ChP relative to expression in all other cell types in the ChP.

Gene	$AvgLog_2FC$	Pct. 1	Pct. 2
Gm11730	9.63808643	0.207	0.001
Syt15	9.20110407	0.166	0.001
Gpihbp1	8.93608164	0.563	0.01
Myct1	8.84389601	0.736	0.009
Sox18	8.75487989	0.343	0.003
Unc45b	8.73926407	0.262	0.001
Ccm2l	8.68129412	0.2	0.001
Fam167b	8.59931628	0.669	0.011
Ssu2	8.58850203	0.132	0.001
Tmem252	8.52942732	0.261	0.002

$AvgLog_2FC$ provides an index of the expression of EC marker genes relative to the expression of these genes in all other cell types. Percentage 1 (Pct. 1) shows the fraction of ECs within the ChP that express EC marker genes; percentage 2 (Pct. 2) shows the fraction of non-EC cell types that express the EC marker gene.

Table S2. RT-PCR primers.

**Targets**	**Forward (5′→3′)**	**Reverse (5′→3′)**
Mouse *Gapdh*	TCAAGAAGGTGGTGAAGCAGG	TGGAAGAGTGGGAGTTGCTGT
Mouse *Gpihbp1*	AGCAGGGACAGAGCACCTCT	AGACGAGCGTGATGCAGAAG
Mouse *Pecam1*	CCAAAGCCAGTAGCATCATGGTC	GGATGGTGAAGTTGGCTACAGG
Mouse *Folr1*	GGACTGAACTTCTCAATGTCTGC	CTTCCTGGCTTGTGTTCGTGGA
Mouse *Ttr*	GAGTAGAACTGGACACCAAATCG	CTGCGATGGTGTAGTGGCGATG
Human *GPIHBP*	GATGACTACGACGAGGAAGATGAGG	GAGCAGTTCTGCGTCAGGTTGC
Human *LPL*	TAGCTGGTCAGACTGGTGGA	TTCACAAATACCGCAGGTG
Human *PECAM1*	GGAGTGATCATTGCTCTCTTGAT	TTGGAGTTCAGAAGTGGTACTGC
Human *GAPDH*	GCTCTCTGCTCCTCCTGTTC	ACGACCAAATCCGTTGACTC

References for Supplementary Figures

1. Dani N, Herbst RH, McCabe C, Green GS, Kaiser K, Head JP, et al. A cellular and spatial map of the choroid plexus across brain ventricles and ages. *Cell.* 2021;184(11):3056-74. e21.

2. Pfau SJ, Langen UH, Fisher TM, Prakash I, Nagpurwala F, Lozoya RA, et al. Characteristics of blood-brain barrier heterogeneity between brain regions revealed by profiling vascular and perivascular cells. *Nat Neurosci.* 2024;27(10):1892-903.

3. Hill AD, Okonechnikov K, Herr MK, Thomas C, Thongjuea S, Hasselblatt M, et al. Single-nucleus RNA-seq dissection of choroid plexus tumor cell heterogeneity. *EMBO J.* 2024;43(24):6766-91.
